# Production-Ready Double-Sided Fabrication of Dual-Band Infrared Metaoptics Using Deep-Ultraviolet Lithography

**DOI:** 10.1021/acsnano.5c11908

**Published:** 2025-10-19

**Authors:** Kai Sun, Xingzhao Yan, Jordan Scott, Jun-Yu Ou, James N. Monks, Otto L. Muskens

**Affiliations:** 1 Physics and Astronomy, Faculty of Engineering and Physical Sciences, 7423 University of Southampton, Southampton SO17 1BJ, United Kingdom; 2 Optoelectronics Research Centre, Faculty of Engineering and Physical Sciences, 7423 University of Southampton, Southampton SO17 1BJ, United Kingdom; 3 Teledyne Qioptiq Ltd., Glascoed Rd, Saint Asaph LL17 0LL, United Kingdom

**Keywords:** metaoptics, metalens, deep-UV lithography, multiband, LWIR, MWIR, thermal imaging

## Abstract

Metaoptics, the application of metasurfaces into optical systems, is seeing an accelerating development owing to advantages in size, weight, and cost and the ability to program optical functions beyond traditional refractive optics. The transition of metaoptics from the laboratory into applications is enabled by scalable production methods based on highly reproducible semiconductor process technology. Here, we introduce a method for the fabrication of double-sided metasurfaces through deep-UV lithography as a production-ready method for achieving high-quality metaoptics. We achieve patterning of a silicon wafer on both sides with mutual alignment of around 10 μm based on tool accuracy without requiring through-wafer alignment markers other than the wafer notch. An application highlighting the benefits of double-sided design is demonstrated in the form of a dual-band metalens with independent control over focal lengths in mid- and long-wavelength infrared bands. Using multireticle stitching, we demonstrate a 40 mm diameter, large-area metalens with excellent broadband imaging performance, showing partial canceling of chromatic dispersion when used in a hybrid configuration with a BaF_2_ refractive lens. Our work establishes a production-ready approach to infrared metaoptics designs and double-sided metaoptics fabrication with direct potential for translation into scalable technology for real-world applications.

## Graphical abstract

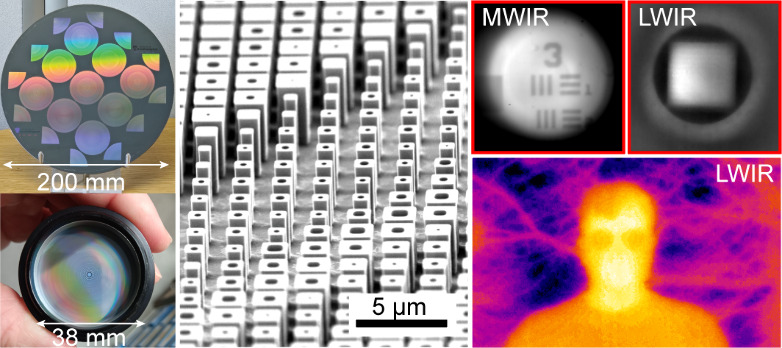

Metasurfaces consisting of subwavelength nanostructures allow for engineering of optical responses beyond that of the bulk material properties including amplitude, phase, polarization, and absorption.^1–6^ Metasurface optics, or in short metaoptics, have gained significant attention with research progress in recent years and are commonly referred to as ‘flat optics’ for the thickness being essentially that of the nanostructured layer and its substrate, which remains unchanged with increasing diameter. Metaoptics including metalenses could be highly desirable over bulk refractive solutions in terms of size and weight, materials selection, thermal, and mechanical stability,^7^ which are critical parameters in applications such as space and low-form factor platforms such as smartphones and wearables. In the past decade, metaoptics have been intensively investigated in the visible range^8–12^ and near-infrared and short-wave infrared (SWIR) bands^13–17^ for potential applications in a wide range of areas including AR/VR, 3D imaging, facial scanning, lidar, and astronomical telescopes, to name a few. Metalenses in this spectral range are mainly formed through layers of amorphous silicon, gallium nitride, or titanium dioxide nanostructures on transparent substrates, e.g., glass/quartz and sapphire, with the nanostructure fabrication as the main challenge.

While most metaoptics laboratory demonstrations were made by using electron-beam lithography^15,18,19^ and two-photon laser writing,^20,21^ increasing progress has been made on the use of scaled-up fabrication techniques such as deep-ultraviolet (DUV) lithography^17,22,23^ and nanoimprint lithography.^24–26^ The latest developments show promising trends, such as the increase in metalens diameters from a few tens of micrometers^27^ to 100 mm through a multiexposure stitching technique,^28^ while the substrate size is also increased from a few mm chip to 8 in. and even 12 in. wafers in 2024.^22,23^ Advanced functions such as multiband and multiwavelength operation were also reported to further increase the metalens capabilities.^9,29–31^ Double-sided metalenses were investigated with one metasurface on each side of the substrates for their improved capability and performance.^8,12,32–34^ Unlike the single-sided metalenses, double-sided metalenses involve further challenges in ensuring that defined nanostructures are preserved through the subsequent lithography steps. Currently, the demonstrated works only include small-diameter visible and SWIR metalenses fabricated through e-beam lithography, as the DUV lithography offers little flexibility in handling nanostructures as the backside and also has strict substrate requirements, e.g., thickness variation around 1 μm or below, over a 200 mm substrate, to achieve the specified minimal feature controls.

Compared with the decade-long investigation on visible and SWIR metalenses, research focusing on long-wave infrared (LWIR) metaoptics, based on strong theoretical and experimental foundations,^5,35–37^ has seen its major developments in recent years.^36,38–50^ These demonstrations are done mainly using all-silicon implementations, owing to the high refractive index and low absorption in LWIR bands, and matured semiconductor processing methods using e-beam lithography, laser writing, and DUV lithography. At long wavelengths, the nanostructures or pillars are a few micrometers in height, and thus DUV lithography might be a more suitable technique over nanoimprinting for scale-up manufacture. Large-area LWIR metalenses up to 40 mm have only recently been reported through direct laser writing,^40^ while midwave infrared (MWIR) metalenses so far have received limited attention.^51–56^ The midwave band is currently less widely commercially developed, and imaging systems are still high in cost although substantial progress is being made in the development of advanced technologies for MWIR detectors. From a fabrication perspective, midwave metaoptics requires a combination of periodicities and feature sizes that are typically smaller than those achieved using contact i-line photolithography, while the required feature heights are still on the order of micrometers, posing challenges in terms of etch depth and required resist/hard mask thickness for techniques such as nanoimprint, which generally work with thinner resists. A combined MWIR and LWIR dual-band imaging system can enable operation in diverse environments.^57^ An overview of the recent literature with parameters relevant to this work is presented in Table S1, Supporting Information.

In this work, we demonstrate a wafer-scale double-sided metasurface fabrication method on 200 mm silicon substrates, with no involvement of organic passivation on defined nanostructures and thus little impact on wafer thickness variation, offering full compatibility with DUV lithography and automated wafer coater and developer, commonly referred to as the Track. Based on our proposed double-sided fabrication method, we have developed MWIR/LWIR dual-band metalenses with operation wavelengths of 4 and 10 μm with independent control of the focal length at each wavelength. We further demonstrate that the double-sided process is compatible with size scale-up through a multiexposure stitching technique and we have successfully achieved a metalens with diameter of 40 mm. This technique has been applied to present large-diameter MWIR/LWIR dual-band and LWIR single-band metalenses and to show their real-world performance.

Large-diameter infrared metalenses manufactured through scale-up production technologies are urgently needed to advance the emerging infrared imaging sector. Infrared imaging is gaining significant popularity for its increasingly broad application in sectors including autonomous driving, road safety, industrial monitoring, gas sensing, medical imaging, building energy efficiency, wildlife monitoring, satellite earth observation, defense, security, and space applications. The LWIR and MWIR bands are two critical atmospheric transmission windows, which correspond to the near-room-temperature blackbody emission peak for LWIR and the higher temperature emission for MWIR, as well as absorption wavelengths of several gases, e.g., methane (CH_4_), nitric oxide (N_2_O), and carbon dioxide (CO_2_). For LWIR optics, most classical optical materials such as oxide glass and polymers are opaque due to phonon absorption, leaving the conventional solutions to single-crystal germanium and chalcogenide glasses such as zinc sulfide (ZnS) and zinc selenide (ZnSe). These materials either are high-cost and hard to source (Ge) or have limited mechanical robustness (ZnS and ZnSe). For MWIR optics, there are similar material challenges. Moreover, Ge lenses suffer from the thermo-optical focal drift, and ZnS and ZnSe lenses have limited thermal shock resistance and are fragile against scratching.

Metaoptics could offer a cost-effective alternative able to address some of the open challenges in infrared imaging technology. Our choice of dual-band metaoptics is motivated by an interest of covering both technologically relevant infrared bands from 3 to 5 and 8 to 12 μm. The wavelengths of 4 and 10 μm were chosen to be the center of those bands. The wide separation in wavelengths is technologically challenging for conventional optics, and advanced metasurface concepts based on multiscale geometries with low cross-talk offer an interesting avenue for exploration. This avenue is enabled by the technological advances presented in this work, allowing reliable fabrication over multiple length scales. The work in this study offers a number of promising opportunities for double-sided infrared metaoptics.

## 2 Results and Discussion

### 2.1 Fabrication of Double-Sided Metaoptics Using Deep-UV Lithography

To achieve the production-ready fabrication of double-sided meta-optics, the key challenge is to ensure its compatibility with automatic handling through DUV lithography and Track systems. The proposed method is schematically shown in Figure 1a, with the key element being the use of a double-hard masking method. The process flow starts with a sub-1 μm SiO_2_ layer growth on both sides of double-sided polished (DSP) silicon substrates through thermal oxidation (step 1). The DUV lithography was subsequently performed on one substrate side referred to as front side (step 2). After the lithography, the SiO_2_ layer is plasma etched as the hard mask for subsequent Si etching (step 3). After the resist removal, the wafer is flipped over with patterned Front side down and the second DUV lithography is performed on the other substrate side referred as back side (step 4).

**Figure 1:**
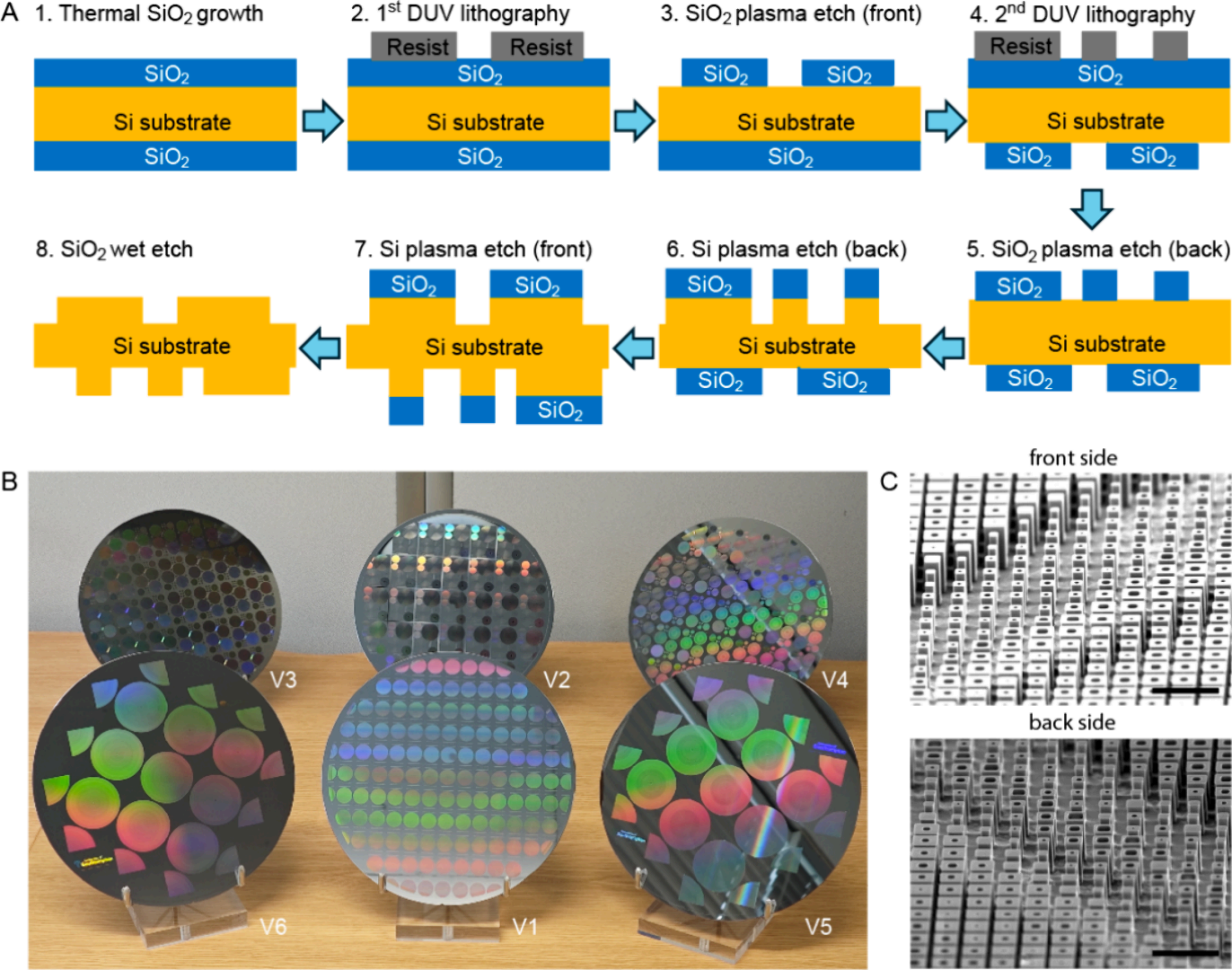
Wafer-scale manufacturing of double-sided metaoptics through deep-UV. (a) Process workflow for fabrication of double-sided metaoptics using deep-UV lithography and plasma etching. (b) Family photograph of six generations of metaoptics fabricated on 200 mm silicon wafers labeled V1–6. (c) SEM images taken at 65° sample tilt from representative arrays at front and back of wafer V5. Scale bars, 5 μm.

Here, the SiO_2_ layer thickness is crucially chosen to be below 1 μm, which makes patterned SiO_2_ structures consistent with the unpolished side of standard silicon wafers with a typical roughness below 1 μm. The DUV lithography and Track systems are capable of handling the patterned SiO_2_ side directly without any modification. The front-to-back side alignment is handled by the DUV lithography system through substrate notch identification. The back side of SiO_2_ is then also patterned using the same plasma etching (step 5). After this step, the most critical lithography steps were completed with the hard mask SiO_2_ layers on both sides well-patterned. Subsequently, the patterns are transferred from the SiO_2_ hard mask into the Si substrate through plasma etching in two steps: one for back side (step 6) and one for front side (step 7). After the Si etching, the SiO_2_ hard mask on both sides is removed through a wet etch in buffered hydrogen fluoride (HF), leaving a double-sided metasurface over Si substrates (step 8). The proposed method is capable of manufacturing large-diameter metalenses beyond the exposure size limit (33 × 25 mm^2^) of the DUV lithography tool used (Nikon NSR-204B scanner, 248 nm KrF DUV wavelength) through a multiexposure stitching step over predefined alignment marks (Section S2, Supporting Information).

Figure 1b shows a photograph of the first six fabricated double-sided substrates by our team with meta-optics of different diameters and functions. The fabricated metalenses have diameters ranging from a few millimeters up to 40 mm. Results from wafers V4–V6 in particular will be discussed in this work. Scanning electron microscopy (SEM) images of the front and back sides after processing are shown in Figure 1c. In this particular design, hollow pillars were used with minimum feature sizes of around 200 nm, given by the 248 nm DUV fabrication process. The etch depth on both sides was nominally 3.5 μm, with some variation dependent on gap size due to microloading effect (Section S3, Supporting Information). This etch depth meets MWIR band requirements even for single-sided metaoptics, while the double-sided metaoptics geometry also ensures that total etch depths of 7.0 μm match the requirement for phase control in the LWIR band.

In this method, the accuracy of registration of features on both sides of the substrate is defined by the mechanical accuracy of the tool, with the only through-wafer marker being the wafer notch provided by the substrate manufacturer. Accuracy and reproducibility of the wafer notch, in combination with notch recognition features on the DUV tool, are therefore critical for obtaining the alignment of the double-sided metaoptics components. Alignment measurement patterns were included on the DUV reticles, which allow verification of registration after the fabrication using an infrared camera. In particular, we found that a rotational error of the wafer upon flipping results in a misalignment that is increasing away from the center of the wafer, and best results were obtained for lenses in the center of the wafer. It appears possible to calibrate out the rotational misalignment within a given batch of wafers by applying an extra wafer rotation to the second exposure. Results (Section S4, Supporting Information) indicate that an absolute alignment error of <10 μm in *X*, 0 μm in *Y*, and 0.01° in wafer rotation between front and back side could be achieved for wafer V5 by including the rotational correction. A 10 μm misalignment of front-to-back side amounts to 1.4% of the 725 μm substrate thickness, which might be considered as a skewness of the double-sided metaoptic with respect to the optical axis of 0.8°.

### 2.2 Design of Dual-Band Metaoptics

To demonstrate the functionality of our double-sided metaoptics platform, we target here the design and fabrication of a dual-band metalens operating simultaneously in MWIR and LWIR bands. This functionality is achieved by a joint optimization of the two metasurfaces, which is computationally implemented using a commercial simulation platform (RSoft Metaoptics Designer,^58^ Synopsys) using a bidirectional scattering distribution function (BSDF) database generated by using the rigorous coupled wave analysis (RCWA). In our demonstration, the pillar height is limited by the available etch depth of around 4 μm in our DUV fabrication workflow using plasma (ICP) etching. This height limits the available phase that can be generated using a single metasurface at LWIR to below 2π coverage, thus requiring the use of a double-sided metasurface to define one metalens. Two design wavelengths were selected as 4 μm for MWIR and 10 μm for LWIR. Unit cell periods between 1.8 and 2.6 μm were tested and a period of 2.2 μm was found to offer a good compromise of performance at both wavelengths.

As a basic design concept, we use two independent length scales in a single unit cell to control the two different wavebands. Symmetric structures such as hollow squares and crosses allow polarization-independent performance of the metalens. Compared to crosses, hollow squares furthermore provide two short-axis resonators within each unit cell, reducing the diffractive losses in the MWIR band. A crossover from hollow squares to filled squares and crosses was defined in a simple parametrization using four rectangular segments of length *L* and width *W* placed at offset

$$\Delta_{x,y}=\frac{L}{2}-\frac{W}{2}$$ 1

where Δ_*x*,*y*_ denotes the offset in either *x* or *y* as shown in Figure 2a. We vary *L* from 0.2–1.8 μm and *W* from 0.2–0.9 μm with a fixed pillar height of 3.5 μm, resulting in the maps shown in Figure 2b,c at 4 and 10 μm wavelength for transmission and phase for periodic metasurface arrays on a single silicon-air interface. The phase map at 4 μm spans multiple orders of 2π, showing a large variation in available design conditions even when using a single metasurface layer. While such a large phase diversity is needed to achieve independent control over the optical phase within the two bands, strong variations will increase sensitivity to fabrication tolerances, resulting in increased wavelength dependence of performance. We also observe that there are considerable regions of parameter space where transmission is low at a 4 μm wavelength, in particular for the larger filled squares due to a breakdown of the design principles requiring at least one small length scale compared to the wavelength. At 10 μm wavelength, transmission is overall high ranging between 85% and 96%, while the optical phase covers roughly half of the required full 2π range, thus necessitating the use of two metasurface sides to achieve its full optical functionality.

**Figure 2:**
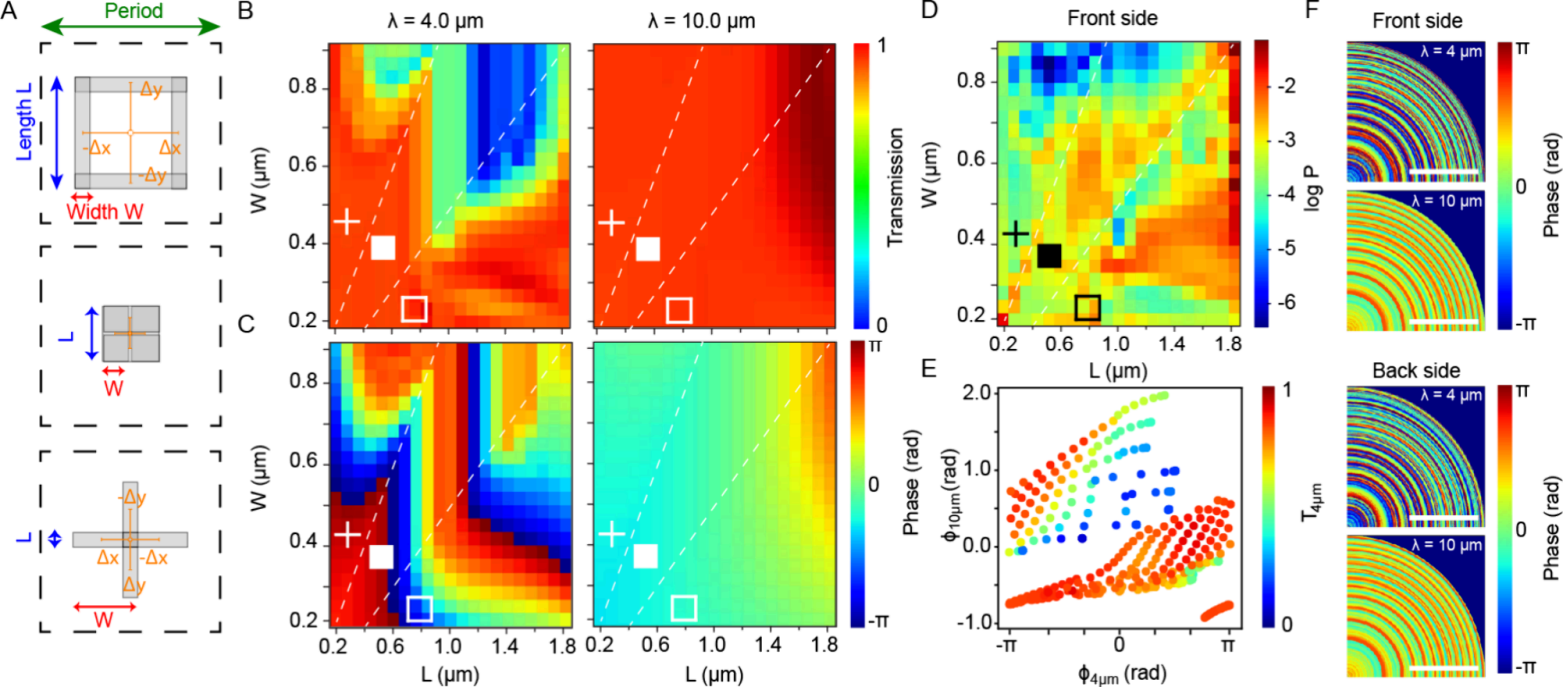
Design of double-sided dual-band metalenses operating at λ = 4 and 10 μm. (A) Definition of design space with period *P*, length *L*, and width *W* and transition from hollow and filled squares to cross shapes following eq 1. (B) Design space showing transmission and phase response at two wavelengths λ of 4 and 10 μm. Dashed lines indicate transitions between shapes. (C) Probability (log-scale) for selection of each design element in the large-area dual-band metalens (V5). (D, E) Parametric plot of phase ϕ_4 μm_ and ϕ_10 μm_ for the design space. (F) Example phase profiles (quadrant) for small-diameter (2 mm) F/2 lens showing generated front and back side profiles at λ = 4 and 10 μm. Scale bars: 0.5 mm.

As the metasurface optimization prioritizes lens transmission against desired point spread function at both wavelengths, it should converge toward regions with higher transmission within a certain penalty range for losing the ideal phase response. An example of this is shown in Figure 2d, which plots the probability P for each element in the database to be selected in a 40 mm diameter dual-band lens with 3.2 × 10^8^ elements. Results are shown for the front side, with very similar results obtained for the back side (Figure S9, Supporting Information). Overall, the optimization algorithm selected predominantly hollow squares (56.6%) followed by filled squares (41.1%) and only very few crosses (2.3%). Areas with higher probability and those that are mostly avoided can be located in the parameter map. Larger rings located on the right-hand side of the parameter space are often required in the design to access the higher optical phase shifts for LWIR, at the cost of some MWIR transmission.

The diversity of elements selected in the optimization process is indicative that the dual-band design requires substantial design space in order to satisfy simultaneously phase combinations at both wavelengths. A different way of presentation, this space is through a parametric scatter plot of the optical phases ϕ_4 μm_ and ϕ_10 μm_ for each element, as shown in Figure 2e. Here, we see that for a single metasurface, indeed, a wide range of combinations for ϕ_4 μm_ and ϕ_10 μm_ can be addressed. Each data point is furthermore color coded according to their transmission at 4 μm wavelength, to illustrate the regions of low MWIR transmission (blue data points). Compared to the single metasurface, double-sided metaoptics allow different combinations of phases to be constituted by choosing appropriate linear combinations of phase elements for each side, taking into account the wave propagation inside the substrate, thus offering a much wider range of choices for the dual-band design than for a single-sided metaoptic device. A typical example of a double-sided optimized design is shown in Figure 2f showing the resulting optical phase profiles at 4 and 10 μm wavelengths, for a small 2 mm diameter lens. The small diameter was chosen to allow resolving the individual rings; however, results are very similar for much larger lens diameters. The phase profile at 10 μm resembles that of a traditional lens with a hyperbolic phase profile and approximately half the required phase on each side, while the profile at 4 μm shows much more rings with varying phases that cannot be easily traced back to a particular functional shape.

### 2.3 Fabrication of Dual-Band Metalenses with Independent Focal Length Control

Using the metasurface database of Section 2.2, we proceed with developing a set of dual-band metalenses with diameters of 12.5 mm. Three different designs are compared, where we select a fixed focal length of 25 mm (f-number F/2) at 4 μm wavelength and focal lengths of 20, 25, and 30 mm at 10 μm wavelength, resulting in f-numbers of F/1.6, F/2.0, and F/2.4, respectively. Figure 3a illustrates the design concept with the three LWIR focus conditions color coded in blue, green, and red. The different lens designs are all accommodated on a single 25 × 33 mm^2^ reticle to allow fabrication on the same wafer, thus eliminating any variation in fabrication parameters between designs. Figure 3b shows the front and back sides of the fabricated wafer, containing >100 individual metalenses.

**Figure 3:**
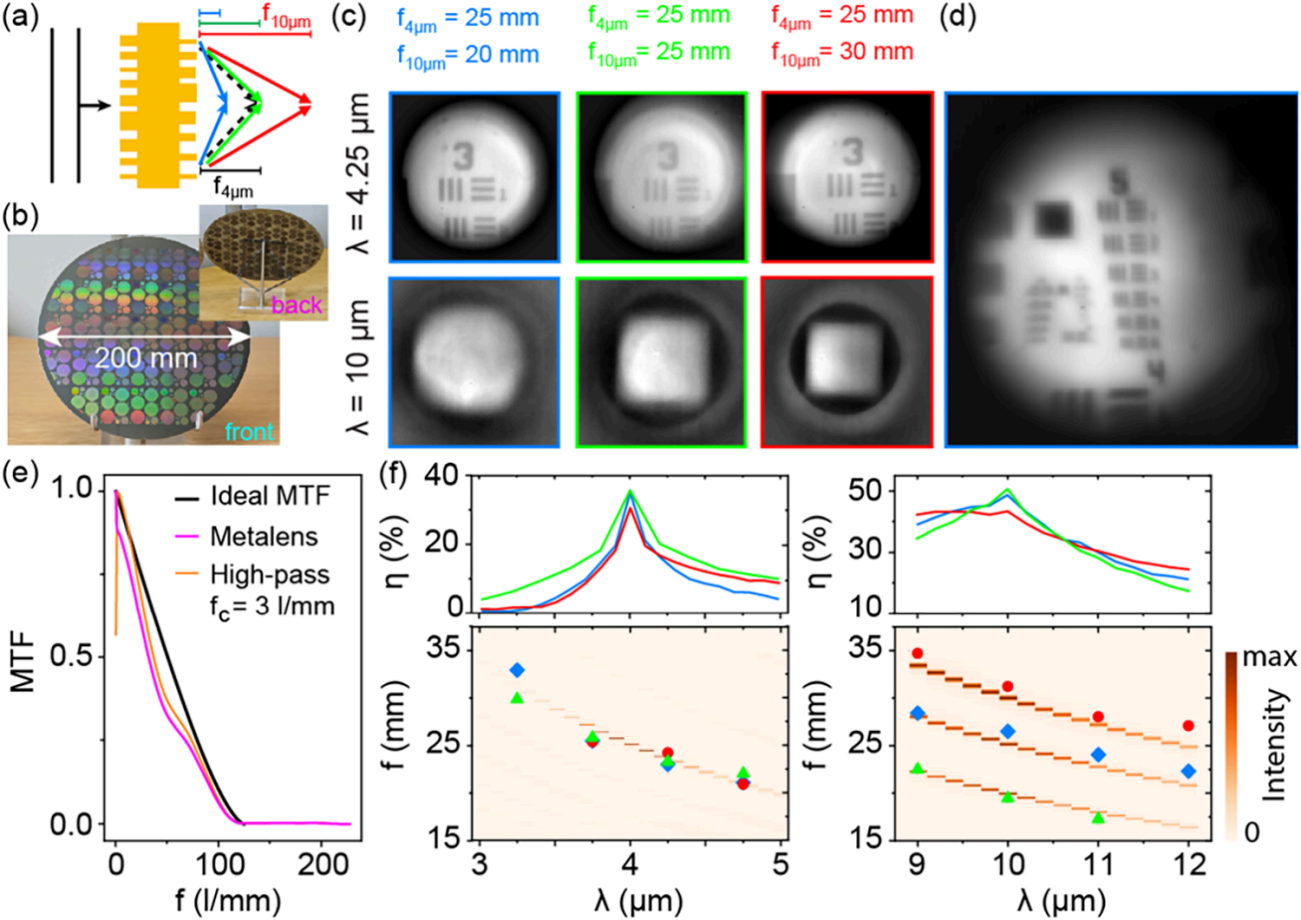
Optical characterizations of the fabricated half-inch metalenses. (a) Schematic illustration of concept of dual-band metaoptics with independent control over focal lengths in midwave and long-wave bands. (b) Photograph of front side of double-sided metaoptics wafer, with the back side shown in inset. One wafer contains 110 individual lenses of 12.5 mm diameter. (c) Optical characterization of three metalenses with the same design focal length at λ = 4.25 μm and different focal lengths at long-wave λ = 10 μm using a 1951 USAF test target (group 3) and MEMS light source as objects for imaging. (d) Image of USAF group 5 using the same configuration as (c) at λ = 4.25 μm. (e) Simulated MTF for design metalens (magenta) compared to ideal (aberration-free) MTF (black) at λ = 4 μm. Effect of high-pass filtering at 3 lines/mm cutoff frequency to reduce haze background is shown by orange curve. (f) Experimentally measured (symbols) focal distances compared to simulated intensities (red color maps) at midwave and long-wave bands. Top panels in (e) are simulated focus efficiencies η within a 52.8 μm diameter around the focus.

Focal lengths in the MWIR range were determined by using a 1951 USAF positive test target on glass in a reverse configuration, where the metaoptic was used as an imaging objective and the achromatic camera lens as a tube lens. As the illumination source, light from a silicon nitride membrane blackbody source at a temperature of 450 °C was spectrally filtered using a set of bandpass filters with center wavelengths of 3.25, 3.75, 4.25, and 4.75 μm and bandwidth of each filter of 0.5 μm. Resulting images at λ = 4.25 μm of group number 3 (elements 1 and 2) are shown in Figure 3c, with an image of group 5 elements shown in Figure 3d showing the capability of imaging at up to 50 lines per mm (element 6), limited by the resolution of the InSb camera. Some blurring is caused by the finite filter bandwidth.

A high-frequency enhancing filter is applied to all images in this study as a very simple haze correction requiring no prior knowledge of the metalens (Section S6, Supporting Information). The filter suppresses lower spatial frequency components below 3 lines per mm; an illustration of this is shown in Figure 3e for simulated MTFs obtained by Fourier transformation of the point spread function at the focal plane. The filtering reduces contributions from zero-order transmission and chromatic aberration-induced blurring. More advanced dehazing algorithms may be implemented beyond the scope of this demonstration.^59^ The simulated MTFs show a higher contrast than experimental images, which can be caused by several experimental artifacts, most notably multiple reflections inside the silicon wafer and other multiple scattering contributions, chromatic dispersion over the 0.5 μm spectral bandwidth, and nonideal backlight illumination using the blackbody source.

Focal lengths at LWIR were determined by direct imaging of the surface of the 2.1 × 1.8 mm^2^ SiN light source itself, as the USAF substrate did not transmit sufficient light to enable its use at longer wavelengths. Bandpass filters were used with center wavelengths of 9, 10, 11, and 12 μm and bandwidth of 0.5 μm, and results for 10 μm are shown in Figure 3c. Other results were taken for the different spectral filters (Section S7, Supporting Information), resulting in values of the focal length *f* against wavelength shown in Figure 3f (data points).

Clearly, the three different lenses produce different focal lengths in the LWIR and therefore different magnifications of the test image, whereas the focal length at the MWIR remains unchanged. This experimental evidence confirms that the lenses successfully operate as dual-band MWIR/LWIR optical elements with independent control over the focal length, resulting in good-quality images in both bands. We can directly compare these results against the model design by plotting the simulated on-axis intensity against distance *z* as a color map for each evaluated wavelength. Here, the color scale indicates the intensity normalized against the maximum in each band. Simulation results for the three different focal lengths in LWIR are well separated and could be overlaid for visualization purposes; separate results are shown in Section S7, Supporting Information. Absolute focusing efficiencies η within are also extracted from the simulations using a 50 μm diameter aperture located at the distance corresponding to the maximum on-axis intensity for each wavelength and are plotted in the top panels of Figure 3e. We see that the designed lenses achieve a peak efficiency of around 35% at λ = 4 μm with a spectral bandwidth of ± 0.25 μm around this maximum. In comparison, the efficiency at LWIR is higher and reaches up to η = 50% at 10 μm with a spectral band exceeding ± 1 μm around the maximum. Apart from the efficiency drop, we also observe the 1/ λ wavelength dependence of the focal length expected for diffractive optical elements without chromatic correction.

To measure the absolute focusing efficiency, a CO_2_ laser at 10.8 μm wavelength was used in combination with a 200 μm pinhole (Figure S17, Section S7, Supporting Information). All three lenses show an absolute transmission of 46% and relative focus efficiency of between 50% and 55%, yielding absolute focus efficiencies of 23%–26%. Numerical design simulations in Figure 3e indicate an ideal efficiency of around 30%, which remains largely the same for collection apertures above 50 μm. Therefore, our lenses perform within the expected range of efficiencies at the LWIR.

### 2.4 Large-Area Double-Sided Metaoptics Demonstrators

Following the successful demonstration of double-sided metaoptics at a 12.5 mm diameter, we proceed by increasing the metaoptic diameter to 40 mm. This increase in size requires a multiexposure stitching approach, which was implemented using a single reticle per side as illustrated in Figure 4a; horizontal write fields are indicated by orange and vertical (rotated over 90°) are indicated by green colors. The reticle itself consists of two quadrants of the metaoptic in two opposing corners of the area (Section S2, Supporting Information). Accurate exposure is achieved by aligning with the predefined alignment markers at specific locations on the wafer, facilitating the alignment of the exposure write fields after wafer rotation. In this case, the front-to-back alignment was also through the substrate notch but at the lithography steps for alignment mark definition on both sides. For 40 mm metalens, we have made two designs, one designed for MWIR/LWIR dual-band (V5 in Figure 1b) and the other one for LWIR single band (V6 in Figure 1b). Figure 4b shows the resulting wafer (V5, dual-band) containing 9 individual large-area metalenses. The accuracy of multireticle stitching was found to be less than 100 nm as can be observed in Figure 4c showing a zoomed in SEM of the center of the metaoptic (V5). The central hollow square was segmented into four quadrants, which are matched together well within the edge with of the hollow square, resulting in a well-defined composite structure with only very little underexposure at the edges resulting in very fine (<100 nm) protrusions around the stitched points.

**Figure 4:**
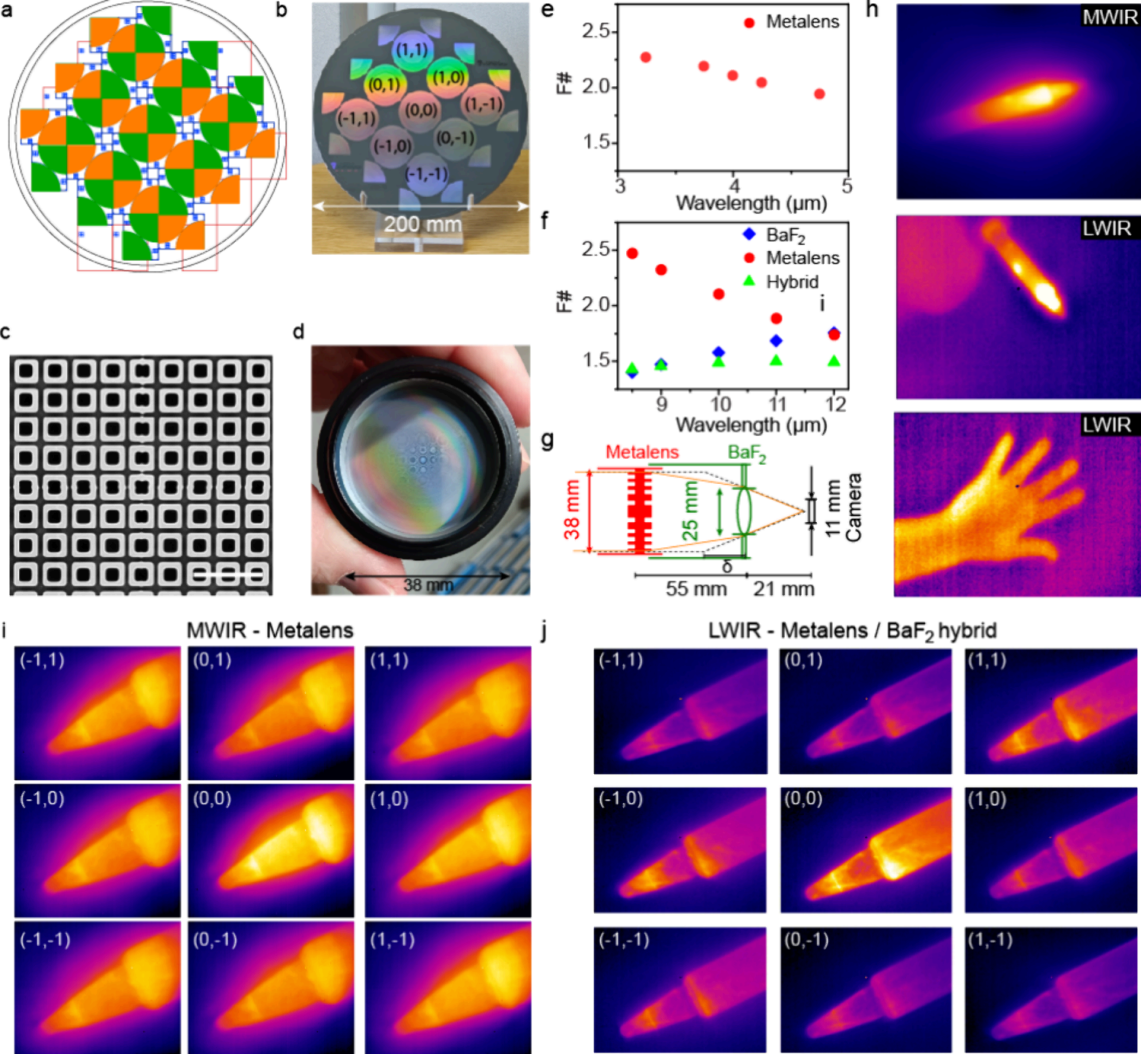
40 mm large-diameter metalenses through multistitching exposure and their real-world thermal imaging performance. (a) Layout of multiexposure stitching with 90° wafer rotation using a single DUV reticle. (b) Photograph of front side of the produced double-sided metaoptics wafer containing 9 complete lenses of 40 mm diameter. (c) SEM image of center of lens showing quality of stitched fields (scale bar, 5 μm). (d) Photograph of metalens laser-cut from the wafer and mounted in optical holder (T-mount) with 38 mm clear aperture. (e, f) F-number against wavelength for MWIR and LWIR. (g) Arrangement of hybrid metalens – refractive BaF_2_ optical system. (h) Example images for soldering iron at 150 °C in MWIR and LWIR and hand at LWIR. Image at MWIR taken using dual-band metalens V5 and LWIR using hybrid BaF_2_ and metaoptic V5 compound. (i, j) Broadband wafer-level characterization of nine dual-band metalenses at MWIR (i) and LWIR (j) bands.

Metaoptics components were laser-cut from the wafer and mounted into individual T-mount lens holders, leaving a 38 mm free aperture, as shown in Figure 4d. Then, the imaging performance can be characterized by mounting the individual metalenses to the commercial MWIR and LWIR cameras as main focusing lenses. These individual metaoptics components can now be readily integrated into commercial MWIR and LWIR optical systems and used for real-world thermal imaging testing. The focal lengths were characterized and are presented in Figure 4e,f as the equivalent F-number for a fixed lens diameter of 38 mm. The strong wavelength dispersion of metaoptics poses challenges for broadband imaging and cannot be easily corrected by lens design for large-diameter metaoptics. In our studies, we make use of the fact that a BaF_2_ refractive lens shows a large chromatic dispersion with opposite sign of the metaoptic (Figure 4f), thus allowing a partial correction of chromatic aberrations while reducing the F-number to around 1.5. The schematic arrangement of the hybrid refractive and meta-optic compounds is indicated in Figure 4g. Here, the 25 mm diameter BaF_2_ lens is the smaller one of the pair and is located closest to the camera, at around 21 mm from the focal plane array. This configuration exploits the short working distance offered by the low-F-number uncooled LWIR camera. The cooled MWIR camera, which has a much longer working distance and a single metalens, was used for the midwave imaging experiments without chromatic correction. Images presented in Figure 4h show examples of imaging performance of the dual-band metalens where a hot soldering iron (150 °C) was imaged in both midwave and long-wave. The image of a hand at a 1.5 m distance shows that the metalens hybrid can image body temperature objects; a direct comparison is presented in Figure S22, Supporting Information, showing how the hybrid compound configuration increases the field of view compared to the individual lenses.

Variations in imaging by the different metalenses across the wafer were characterized by using a wafer-scale setup as described in Section S14, Supporting Information. Figure 4i,j shows images of the tip of a soldering iron (350 °C) for the metalenses, indicating that all lenses are suitable for dual-band imaging. The central lens labeled (0,0) shows the highest transmission which we attribute to the improved alignment due to its significantly lower rotational alignment error; however, intensity variations are within a factor two over the wafer.

Additional laboratory and outdoor bench demonstrations showcasing both the MWIR and LWIR performances are presented in Figure 5. In Figure 5a–h, the target object is a substrate with designed emissivity contrast in the form of the University of Southampton logo of approximately 5 cm in width, positioned at 35 cm from the camera as shown in Figure 5a,b, with further details presented in Section S8, Supporting Information. The target is mounted on a hot plate and heated to 140 °C. The availability of a 40 mm metaoptic allows for a direct like-for-like comparison with the high-specification camera lenses (Janos Nyctea and FLIR, respectively, for mid- and long-wave bands). Figure 5c,f shows the imaging results for these commercial lenses, which represent the ‘ground truth’ due to the superior build and achromatic performance of these multielement lenses. For MWIR, a 2 in. diameter, 3.6 μm long-pass filter was placed in front of the lens to limit the response to the same range as the metaoptic. The LWIR reference image was cropped to an area of interest of F/1.5 (original image F/1) for better comparison with field of view of the metaoptics.

**Figure 5:**
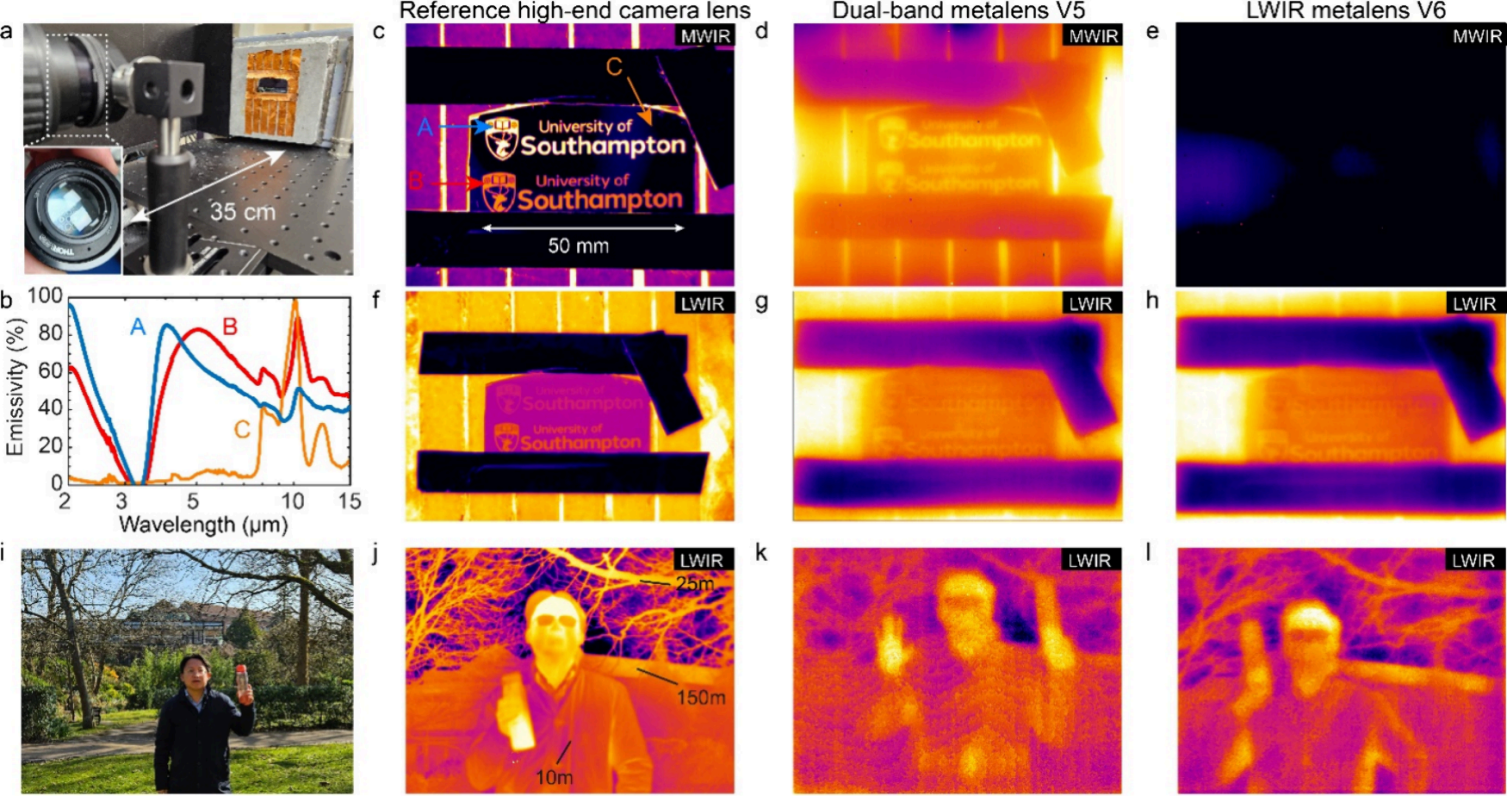
Real-world thermal imaging performance. (a) Experimental arrangement for imaging of a test object at 35 cm distance. (b) Infrared emissivity spectra for the university logo test sample at three locations A, B, C as shown in panel (c). (c–e) Direct comparison of metalens performance against state-of-the-art commercial lens for midwave. (f–h) Performance of hybrid BaF_2_ and metaoptic compound against state-of-the-art commercial lens for long-wave. (f-l) Obtained for university logo target heated to 140 °C. (i–l) Outdoor imaging demonstration using hybrid BaF_2_ and metaoptic compound at long-wave infrared, with details from 10 m and up to 150 m in range. Panels (d), (g), and (k) are for dual-band metalens V5, and panels (e), (h), and (l) are for single-band metalens V6.

In the midwave band, the dual-band metaoptic V5 shown in Figure 5d is able to produce a good-quality image, with letters clearly identified, where some blurring and haze is expected based on the chromatic aberration of the lens. Results for the long-wave optimized metalens V6 are presented in Figure 5e. Clearly, the lack of MWIR functionality results in a complete loss of the MWIR image, which emphasizes the remarkable performance of the dual-band lens in comparison. Corresponding long-wave imaging results are presented in Figure 5g,h for the dual-band and LWIR-only metaoptic, respectively; both were used in a hybrid compound configuration. Again, we can clearly resolve the letters of the print as well as features of the shield logo down to 1 mm. In comparison, images generated using only the metalens (Figure S20, Section S9, Supporting Information) show blurring by chromatic aberration.

Further outdoor testing was done using the long-wave imaging system, as shown in Figure 5i–l. The overall scene (see also Figure S23, Section S11, Supporting Information) shows features at distances of 10 m (person), 25 m (tree branches), and 150 m (roof line). Results for the commercial camera lens in Figure 5j can be directly compared with the results for the dual-band metaoptics (Figure 5k) and LWIR-only design (Figure 5l), both in a hybrid compound configuration. We note an improved performance for the single-band LWIR metaoptic over the dual-band lens as is expected given the trade-off in designing two separate operation wavelengths. In both cases, the features at all three distances can be identified, verifying the operation of the metaoptic up to 150 m range.

The large-area double-sided metaoptics were further characterized using a range of industrial methods commonly used for refractive optical systems, with further details in Section S12, Supporting Information. Results are summarized in Table 1 and include root-mean-square (RMS) wavefront error, peak–valley ratio (PV), Strehl ratio, astigmatism, coma, and spherical aberration at wavelengths of 3.39 and 9.24 μm for MWIR and LWIR, respectively, which were chosen based on available laser sources. For the dual-band metalens, its MWIR performance present a low peak–valley of 0.24 λ and high Strehl ratio of 0.90, which exceed the typical threshold values of <0.25 λ for peak–valley and >0.80 Strehl ratio and therefore indicate that this dual-band metalens is diffraction-limited, that is, its optical performance is governed by fundamental physics rather than imperfections. The RMS wavefront error of 0.051 λ also shows the metalens has minor residual imperfections, which could be from the astigmatism, coma, and spherical aberration. Its LWIR performance also results in a low RMS of 0.042 λ, low peak–valley of 0.28 λ, and high Strehl ratio of 0.93, which are also diffraction-limited. Compared with the dual-band metalens at 9.24 μm, the LWIR metalens gives a lower RMS, lower PV and higher Strehl ratio and also lower astigmatism, coma, and spherical aberration. The observation of superior values for the LWIR metalens matches the observed superior performance of the LWIR metalens over the dual-band metalens in real-world imaging well (Figure 5k,l).

**Table 1:** Metalens Interferometric Characterizations

	dual-band metalens (V5)		LWIR metalens (V6)
wavelength	3.39 μm	9.24 μm	9.24 μm
RMS wavefront error	0.051λ	0.042λ	0.029λ
peak–valley	0.24λ	0.28λ	0.10λ
Strehl ratio	0.90	0.93	0.97
astigmatism	0.56λ, at −18.1°	0.06λ, at −87.1°	0.04λ, at −63.3°
coma	1.02λ at 5.9°	0.27λ at 153°	0.24λ at −132°
spherical aberration	0.06λ	0.30λ	0.26λ

## 3 Discussion

Current studies demonstrate the feasibility of achieving multiband functionalities using a double-sided metasurface. The choice of ICP etching together with the SiO_2_ hard mask sets a limit on the achievable etch depth, which prevented the formation of a full metalens on one side in the LWIR band. In our work, the ICP process was chosen for its ability to achieve small feature sizes with steep slopes, allowing for precise design of dual-band functionalities. In our work, the maximum SiO_2_ thickness as a hard mask was a trade-off between DUV resist thickness and SiO_2_ etching. However, a more selective and thinner hard mask, e.g., Cr, might be adopted for deeper etching. Deeper etching of 8 μm height pillars such as required for single-sided LWIR metaoptics will also be possible using deep reactive ion etching; however, this comes at the cost of increased minimum feature size and scalloping artifacts, which would then limit the extension to multiband optics at shorter wavelengths using this method.

Our demonstration shows that the double-sided approach offers a viable solution, however with some disadvantages related to the limited ability of improving the antireflection properties of the wafer back side and, importantly, the increased susceptibility to misalignment between the two sides. In our DUV, we found that misalignment parallel to the wafer loading is negligible, and thus, rotational and transverse shifts need to be corrected. While some of these may be calibrated out as shown in this work for a systematic rotational error, more precise pattern alignment strategies may also be possible, for example, using through-the-lens back-side alignment available on some DUV systems. Several other doublet metalens demonstrations reported fabrication on both sides of the substrates^8,13,33,34,51,60^ or stacked as two metalens on top of the other on one substrate side with a passivation layer in-between.^12,61,62^ The doublet misalignments for double-sided metalenses in these works were only briefly mentioned and were achieved through a contact UV lithography for back-side alignment^8,13,60^ or a transmission microscope.^33,34^ It would be expected to be a misalignment of a few micrometers in the case of the back-side alignment using a contact photolithography aligner,^8^ which is expected to result in an offset of a few units for visible metalens. Most published works demonstrate excellent doublet metalens performance, even in the presence of some levels of misalignment, and most achieve this at much shorter wavelengths down to the visible range. More systematic exploration of the effects of double-sided alignment on performance of doublet metalenses will be addressed in future investigations to better understand its role in metaoptical systems.

In this work, double-sided metalenses were fabricated to demonstrate the proposed manufacturing technique. However, the current metalenses have not yet achieved the performance level of commercial state-of-the-art optics, as attaining a high achromatic performance and focal efficiency remains a long-term challenge for metalenses. To enhance achromatic performance, one potential solution is to adopt advanced design methodologies, e.g., artificial intelligence (AI)-assisted inverse approach, which can simultaneously account for multiple considerations.^39,50,63,64^ Another strategy is to correct aberration through hybrid configurations, either by combining a metalens with a refractive lens^41,43^ or by employing multiple metalenses^38^ (or double-sided metalenses). Moreover, ongoing metaoptics research should focus on the integration of metaoptics into hybrid imaging systems using a full end-to-end design optimization framework.^44^ With respect to focal efficiency, the improvement may be achieved by mitigating the high refractive index of silicon through the incorporation of conventional antireflective coatings^43,65,66^ or moth-eye metasurfaces. In parallel, efforts could be directed toward fabrication-aware design workflows that incorporate advanced models of the fabricated metasurfaces to further enhance performance.^58^

## 4 Conclusions

In conclusion, we establish a wafer-scale double-sided metaoptic manufacturing process that is fully compatible with an automatic DUV scanning lithography system and Track. Based on this manufacturing method, we demonstrate dual-band MWIR/LWIR metalenses with independent focal length controls. In addition, we have further demonstrated that 40 mm large-diameter double-sided metalenses with dual-band and single-band operations can be fabricated through a multiexposure stitching method to overcome the DUV exposure field limit. Across the whole 8 in. substrate, all nine 40 mm diameter metalenses demonstrated consistent dual-band imaging capability with the best performance obtained for the central lens. The fabricated metalens performance was further demonstrated through their integration into imaging systems for real-world thermal imaging. Interferometry measurements show that these metalens are diffraction-limited, with minimal aberrations and wavefront imperfections.

The proposed double-sided metaoptic manufacturing method offers a pathway for infrared metaoptics designs and scale-up production of double-sided metaoptics manufacturing. The double-sided metaoptics offers an expanded design space for multifunctionality, such as the dual-band presented in this work, or could accommodate moth-eye antireflection coatings, doublets, or aberration meta-correctors. The demonstrated double-sided lithography technique is compatible with other scale-up manufacturing techniques for meta-optics, such as nanoimprint lithography and direct laser writing, which can support designs and structures beyond existing capabilities.

## Experimental Section/Methods

### Metalens Design

Double-sided metalenses were designed using RSoft Photonic Design Tools (Keysight) on a dual-processor Xeon Gold 6148 2.40 GHz workstation with 512 GB RAM. The unit cell was designed using the RSoft CAD environment, and subsequently the bidirectional scattering distribution (BSDF) function was simulated using rigorous coupled wave analysis (RCWA) implemented in the DiffractMOD simulation tool, taking advantage of parallel processing over 32 nodes in the multivariable optimization and scanning tool (MOST). A range of incident angles from 0–60° with steps of 15°, wavelengths from 3.0–5.0 μm in steps of 0.1 μm for MWIR, and 9.0–12.0 μm in steps of 0.2 μm for LWIR were used. The unit cell was modeled using four rectangular silicon pillars equal in size, each pillar with a long axis length ranging from 0.2–1.8 μm and short axis width ranging from 0.2–0.9 μm. The pillars were arranged to form a square or cross shape, depending on the offset parameter and the length to width ratio as illustrated in Figure 2. The unit cell period was varied in a series of simulations from 1.6–2.6 μm and an optimal period of 2.2 μm was found to give best overall performance. The vertical height of the pillar structures was set to 3.5 μm for the dual-band design, as defined by the achievable etch depth in our process. The period and height for the LWIR-only design was chosen as 2.6 and 4.0 μm, respectively, following further optimization for the 10 μm wavelength design in the absence of a MWIR design target and slight improvements of the etch depth made in the experimental process development. Metalenses were designed from the generated BSDF database using the MetaOptics Designer simulation tool, using an on-axis Airy spot focus as the target objective. An intensity-difference metric was chosen with a weighting that was strongly biased toward achieving a high absolute transmission efficiency with less weight given to matching the exact shape of the Airy profile. The simulation used absorbing boundary conditions and took into account local propagation angles inside the double-sided metalens structure. These parameters were found to yield convergence toward near-diffraction-limited spots with corresponding good-quality MTF and an absolute focusing efficiency limited by the available degrees of freedom in the database. A GDS-II output of both sides of the metalens was generated and further processed using KLayout to include alignment markers, SEM bars, a front-to-back misalignment scale, and several other test structures. Deep-UV reticles were manufactured from MacDermid Alpha Electronic Solutions.

### Deep-UV Lithography Fabrication of Metaoptics

Here, the fabrication process flow is for the 40 mm metalens with exposure stitching. The fabrication of metalens started by growing 800 nm of SiO_2_ on double polished 200 mm Si substrates through wet oxidation at 1000 °C. Through a Nikon NSR-S204B DUV scanning system and TEL Track (an automated resist coating and developing system), the alignment mark was defined on the front side of Si substrates and subsequently transferred into the SiO_2_ layer through plasma etching. Subsequently, the alignment marks were also defined on the back side of Si substrates through the same DUV and plasma etching, with the DUV front-to-back alignment done through notch recognition aligning with the defined alignment marks, DUV steps, with and without substrate rotation of 90°. The pattern was transferred into the hard mask SiO_2_ through ICP plasma etching using fluorine chemistry. After the photoresist removal, the back side SiO_2_ hard mask was also processed through the same DUV lithography and plasma etching process. After the initial preparation of the SiO_2_ hard masks on both sides of the wafer, the Si pillar arrays were transferred from the SiO_2_ arrays using an ICP plasma etch with SF_6_ and C_4_F_8_, first on one side and then on the other side. The etch depth was controlled through etching time with SEM images in Figure S3, Supporting Information. After the etching, an O_2_ plasma treatment was performed to remove the polymer formed during the Si etching. Finally, the SiO_2_ hard masks on both sides of the wafer were stripped in a 7:1 buffered hydrofluoric acid (HF).

### Infrared Measurements

Broadband response was measured using a blackbody light source (Axetris EMIRS200) with a 1.8 × 2.1 mm^2^ active area. Measurements of short focal distance metalenses were done in a reverse configuration, where the metalens was used as the imaging objective of a target object. The target was either the EMIRS light source itself or a 1951 USAF target. An achromatic imaging lens matching each specific camera was used to project the collected image onto the camera. For the MWIR range, we used a CEDIP/FLIR Titanium SC7300 camera with 320 × 256 pixels and a pixel pitch of 30 μm. The matching camera lens for the MWIR was a 50 mm focal length Janos Technologies Nyctea F/2.3 lens (Model 40679, wavelength 1.5–5 μm). For the benchtop demonstrator, we used a 3.6 μm, 50 mm diameter, infrared long-pass filter (Edmund Optics) to limit the spectral range. For the LWIR range, we used a FLIR A655sc uncooled camera with 640 × 480 pixels and pixel pitch of 17 μm. The matching camera lens was a 24.6 mm focal length F/1 FLIR lens (Model T197922, 25 °FOV). For wafer-scale characterization, we constructed a setup with full-wafer access using 300 mm long-range motorized stages (Thorlabs) in all three directions. Diagrams of optical setups are shown in Section S7, S8, and S14, Supporting Information.

### Quantitative Testing of Metalens Performance

The MWIR performance in Table 1 was measured by a 3.39 μm wavelength phase-shifting Michelson Interferometer with a default aperture size of 50 mm. The LWIR performance was measured by a longwave Twyman-Green interferometer with a default aperture size of 250 mm, equipped with a CO_2_ gas laser at 9.24 μm. A discussion of these tests is presented in Section S7, Supporting Information.

## Supplementary Material



## Data Availability

Supporting data used in this work is openly available from the University of Southampton repository at doi.org/10.5258/SOTON/D3494.

## Notes

Overview of the recent relevant literature, experimental details on multiexposure lithography, additional SEM images, double-sided alignment study, additional details on numerical design, image processing details, and additional optical characterization studies (PDF)
