# Supplementary material for: Production-Ready Double-Sided Fabrication of Dual-Band Infrared Metaoptics Using Deep-Ultraviolet Lithography
Source: ACS Nano. 2025 Oct 19;19(42):37218–29. doi: 10.1021/acsnano.5c11908 (PMC12574206; doi:10.1021/acsnano.5c11908)
Supplement: Supplementary file 1 [file nn5c11908_si_001.pdf]

Supporting Information for

Production-Ready Double-sided Fabrication of Dual-Band Infrared Metaoptics Using Deep-Ultraviolet Lithography

Kai Sun, Xingzhao Yan, Jordan Scott, Jun-Yu Ou, James N. Monks, and Otto L. Muskens**

Kai Sun, Jordan Scott, Jun-Yu Ou, Otto L. Muskens
Physics and Astronomy, Faculty of Engineering and Physical Sciences, University of Southampton, Southampton, SO17 1BJ, United Kingdom
E-mail: K.Sun@soton.ac.uk, O.Muskens@soton.ac.uk

Xingzhao Yan
Optoelectronics Research Center, Faculty of Engineering and Physical Sciences, University of Southampton, Southampton, SO17 1BJ, United Kingdom

James N. Monks
Teledyne Qioptiq ltd, Glascoed Rd, Saint Asaph, LL17 0LL, United Kingdom

*Corresponding authors. Email: k.sun@soton.ac.uk, o.muskens@soton.ac.uk

S1. Overview of selected publications on metalens fabrication with focus on large-area, dual-band and infrared range

Table S1 Summarized selected metalens works since 2020.

Year	Band	Diameter (mm)	Double-sided	Multi-band	Lithography	Material	Substrate	Substrate Size (inch)	Ref
**2025**	**MWIR /LWIR**	**40**	**Yes**	**Yes**	**DUV**	**Si**	**Si**	**8**	**This work**
**2025**	**LWIR**	**40**	**Yes**		**DUV**	**Si**	**Si**	**8**	**This work**
2025	LWIR	31.6, 63.6			??	c-Si	Si	4	1
2025	Vis.		Yes		EBL	TiO_2	Si		2
2025	LWIR	24, 32			Laser	Si	Si		3
2025	Vis.	2.8			EBL	a-Si	Glass		4
2025	LWIR	20		2-WL	Laser	Si	Si		5
2024	LWIR	50			UV stepper	c-Si	Si	6	6
2024	Vis.	0.5			EBL	a-Si	Glass		7
2024	Vis.	0.6			Laser	Graphene-oxide	Glass		8
2024	MWIR	11.95	Yes		EBL	Si	Si		9
2024	LWIR	12.2			DUV	Ge	Ge	2	10
2024	LWIR	10			Laser	Si	Si		11
2024	NIR	2.5			EBL	a-Si	Glass		12
2024	LWIR	100			Laser	Si	Si		13
2024	LWIR	40			Laser	Si	Si		14
2024	LWIR	6			Laser	Si	Si	4	15
2024	Vis	0.6			2-photon	TiO_2	Glass		16
2024	NIR	1.58			EBL	a-Si	Glass		17
2024	NIR	10			NIL	a-Si:H	Quartz	4	18
2024	Vis.	100			DUV	SiO_2	Glass	6	19
2024	Vis.	5			DUV	a-Si	Glass	12	20
2024	NIR	4			EBL	a-Si	Glass		21
2024	Vis.	0.1			2-photon	polymer	Glass		22
2024	LWIR	6			DUV	Si	Si		23
2024	SWIR	1.28			EBL	c-Si	Sapphire		24
2023	Vis.	0.5		3-WL	EBL	a-Si:H	Glass		25
2023	Vis.	0.2			EBL	TiO_2	Glass		26
2023	Vis.	0.16			EBL	Si	Sapphire		27
2023	Vis.	10			DUV	Polymer	Glass		28
2023	Vis.	6			NIL	SiN	Glass		29
2023	SWIR	0.4			EBL	Si	Si		30
2023	SWIR	0.03*	Yes		EBL	Si	Si		31
2023	LWIR	20*		2-WL	laser	Si	Si	2	32
2023	SWIR	80			DUV	a-Si	Glass	4	33
2022	Vis.	10	Yes		EBL	Si, SiN	Sapphire		34
2022	SWIR	0.5	Yes		EBL	a-Si:H	Glass		35
2022	LWIR	80			Contact	Si	Si	4	36
2022	SWIR	0.04			EBL	Si	SOI		37
2021	Vis./SWIR	0.2		Yes	2-photon	Nanoholes	Glass		38
2021	LWIR	20			laser	Si	Si	4	39
2021	MWIR	0.7			DUV	Ge	Sapphire		40
2021	Vis.	0.5			EBL	a-Si	Glass		41
2021	SWIR	0.6	Yes		EBL	a-Si	Glass		42
2020	NIR		Yes		EBL	a-Si	Glass		43
2020	Vis./SWIR	0.01-0.02		Yes	EBL	TiO_2	Glass		44

*: square shape, WL: wavelength

Glass: Glass stands for fused silica, quartz and glass substrates, a-Si: amorphous silicon, Si: crystalline silicon.

Vis.: visible, NIR: near-infrared, SWIR: short-wave infrared, MWIR: mid-wave infrared, LWIR: long-wave infrared, DUV: deep ultraviolet lithography, EBL: electron beam lithography, 2-photo: two-photon lithography, Contact: contact lithography, NIL: Nanoimprint lithography, UV: ultraviolet.

S2. Multi-exposure stitching

Two reticles were designed with one for front and the other one for back side, as shown in Figure S1. For the front side labelled A, it includes two quarters of a designed 40 mm diameter metalens layout. Ideally, one quarter can be used to achieve full lens through rotation. However, our DUV is configured with a capability of one 90-degree rotation. Here, it is noted that the DUV reticle can only be loaded with one orientation as there are extra patterns on the reticle edge for machine identification. Thus, two quarters are needed to achieve a full-round lens layout. In addition, there are also misalignment measurement patterns and two alignment mark patterns on top-right and bottom-left, which are used to define alignment marks for metalens exposures and its rotation exposures. For the back side labelled B, the metalens patterns are in similar layouts but mirrored.

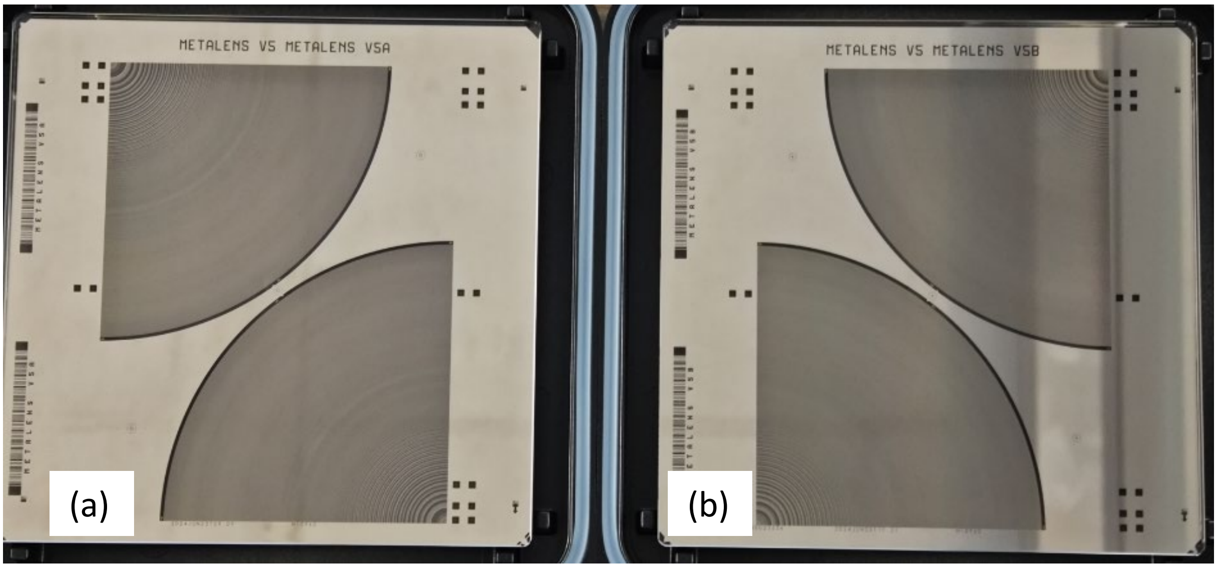

Figure S1 Reticle layouts for multi-exposure metalens designs (40 mm in diameter) (a) front side and (b) back side.

The multi-exposure strategy is illustrated in Figure S2 involving six lithography steps, using the two reticles and substrate rotation functions. Firstly, the alignment mark is defined on the substrate Front side at edge regions as illustrated (Figure S2a) where no metalens will be located on. Secondly, the alignment mark is defined on substrate back side in a mirrored style (Figure S2b), with alignment through wafer notch. Thirdly, metalens layouts (two quarters) are defined at defined locations on the front side (Figure S2c), with alignment control to predefined alignment marks. Fourthly, the other two quarters of metalens layouts are defined using the same reticle but at wafer rotation of 90°, with alignment control also to predefined alignment marks (Figure S2d). Fifthly, the back side layouts are processed in the same

manner, with two quarters defined on back side using back side alignment mark (Figure S2e). Sixthly, the rest two quarters are defined through substrate rotation by 90º (Figure S2f). Here, the design has to be mirrored to reflect the opposite relevant rotation orientation on different sides.

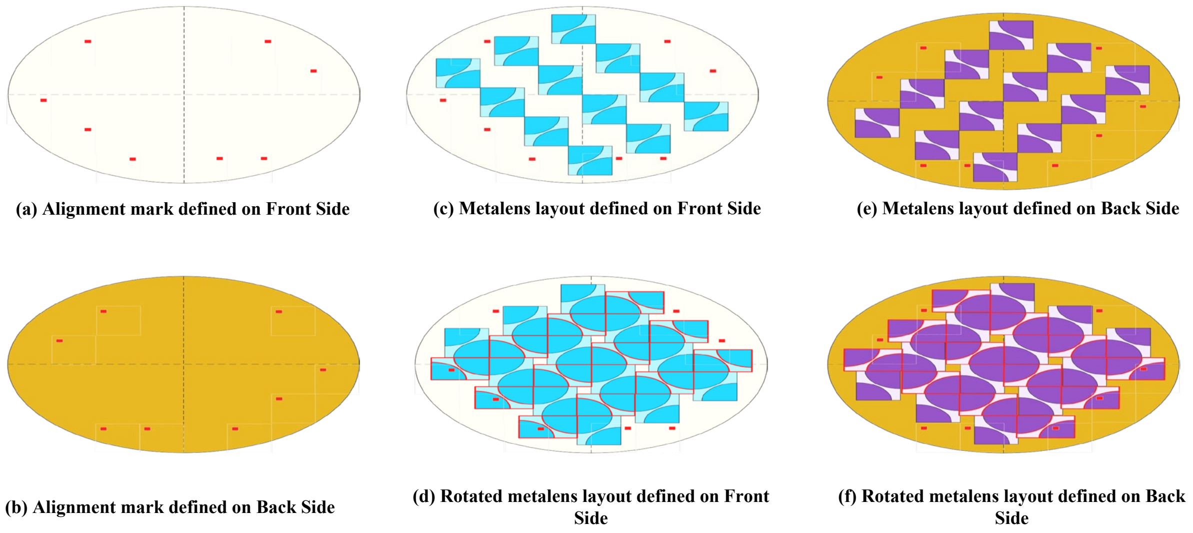

Figure S2 Schematic of multi-exposure stitching process flow. (a) Alignment marks defined on front side of the substrate, (b) Alignment marks defined on back side of the substrate, (c) metalens layout partially exposed on front side and (d) metalens layout partially on the front side with 90o substrate rotation, (e) metalens layout partially exposed on back side, and (f) metalens layout partially exposed on back side with 90o substrate rotation.

S3. Scanning electron microscopy images

Figure S3 presents the cross-sectional SEM images of one etch test wafer with the SiO_2 hard mask on Si pillars. For the front side in Figure S3a, the etch depth varies from 3.24 to 3.64 µm with a mean etch of 3.44 µm. Si pillars (square rings) are seen to be well-defined and vertical. The SiO_2 hard mask layer is about 379 nm left. For the back side in Figure S3b, the etch depth varies from 3.27 to 3.74 µm with a mean etch of 3.50 µm. Si pillars (square rings) are seen to be well-defined and vertical. The SiO_2 hard mask layer is about 390 nm left. Therefore, etch depth on both sides can be well defined through timed etching.

SEM inspections were performed on a metalens as presented in Figure S4, which is from the test wafer given the same etch conditions, with SiO_2 removed but no O_2 plasma treatment given. From these images, the square rings and square pillar are well defined. The central stitching can be identified, indicating some misalignments at the bottom two quadrants in x-axis in a few ten nm but superb for the top two quadrants. There is some ring deformation at the stitching joints and this is attributed to the slight lower exposure at the very edge of the

exposure field. In future work, these ‘joints’ between individual rings can be corrected by slightly modifying the features across the stitching boundaries or shift the feature out of the stitching boundaries. However, the impact is expected to be quite limited.

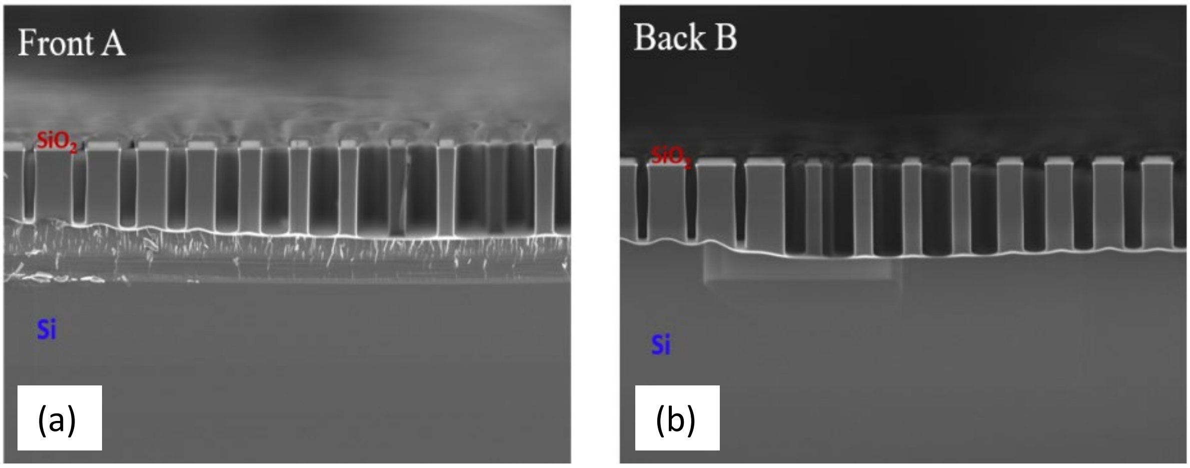

Figure S3 Cross-sectional SEM images of front side (a) and back side (b) of etched Si metalens structures on an etch test wafer for design V5, cleaved through the hollow squares. Etch depth variations with gap size can be seen due to microloading effect.

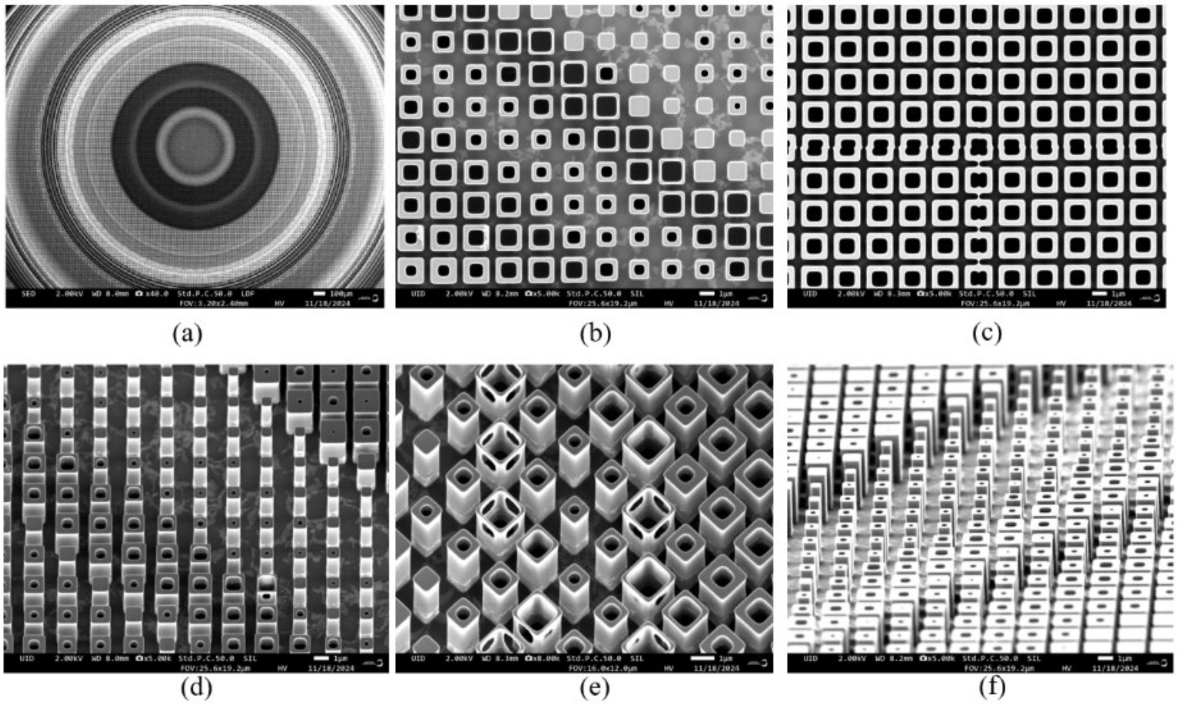

Figure S4 SEM images of the Front side of a 40 mm metalens wafer V5, (a) low-magnification at the metalens center, (b) top-right near-the edge, (c) stitching at the center, (d) 25° tilt, (e) 25° tilt and 25° rotation, and (f) 65° tilt. SEM bars are 100 µm for (a), 1 µm for (b) and 1 µm for (c)-(f).

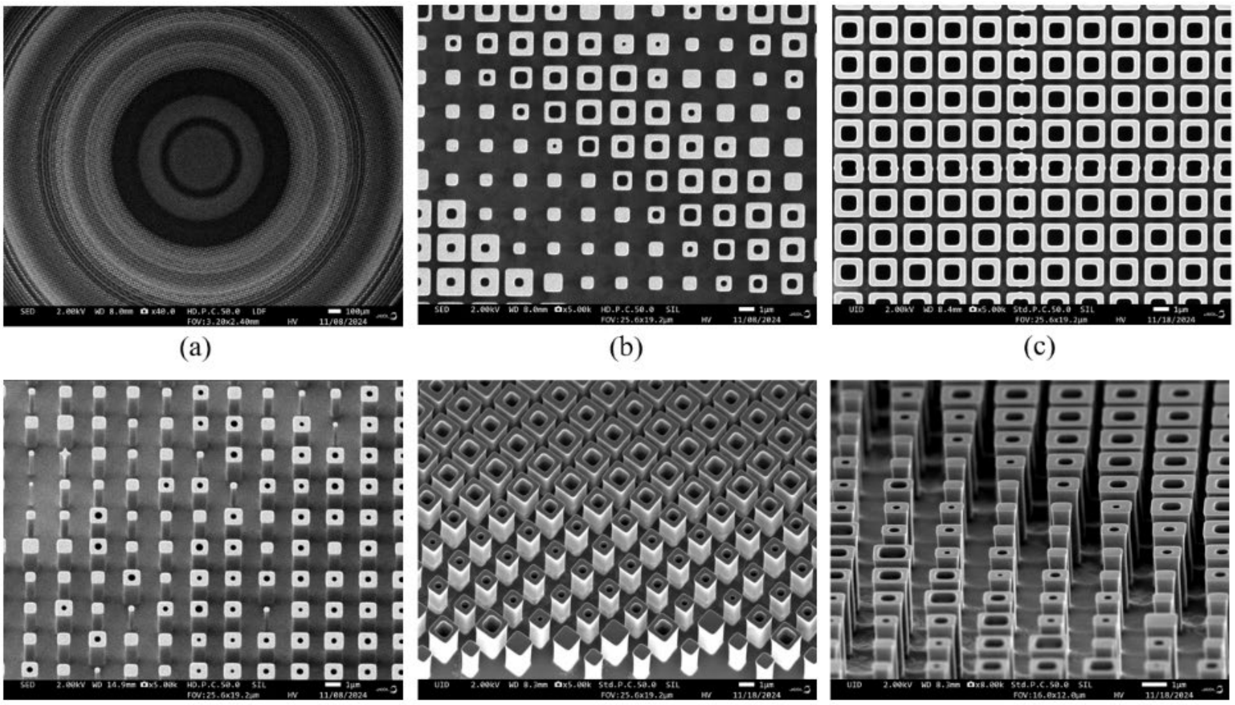

(a) (b) (c)

(d) (e) (f)

Figure S5 SEM images of the Back side of a 40 mm metalens (Batch 3), (a) low-magnification at the metalens center, (b) top-right near-the edge, (c) stitching at the center, (d) 25° tilt, (e) 25° tilt and 25° rotation, and (f) 65° tilt. SEM bars are 100 µm for (a), 1 µm for (b) and 1 µm for (c)-(f).

The images give further insight in the formed structures with smooth sidewalls. It is of interest to see that in some cases holes are formed at the sidewalls of the thinnest square rings, which can be attributed to plasma ion deflection during the etching. In general, the Si etching control is found to be excellent. The results for the back side shown in Figure S5 are similar to those on the front side. In summary, the nanostructures are well-defined including square pillars, cross pillars and square ring pillars, with excellent stitching control.

S4. Pattern registration verification on both sides of wafer

One of the key technical challenges is to align the two metalens sides without dedicated alignment marks as the DUV system is not configured for back side alignment. Instead, the alignment was achieved through wafer notch identification using its own automatic system. We have set alignment measurement patterns on both reticle layers at the center of exposure field as in Figure S6a. It has a vernier scale for 4 µm misalignments (4 units) on both X and Y directions. For misalignment above 4 µm, it can be estimated directly through the center pattern shifts. The misalignment measurement was done through our MWIR camera as the silicon substrate is transparent in this range. As the wafer is manually mounted into the

camera system some global rotation of the marker fields is observed in most of the images which does not relate to the front-back misalignment. Figures S6b and S6c shows typical IR images of misalignment patterns taken at focus of Front A and Back B, respectively. The misalignment can be most easily read out by looking at the relative positions of the two front and back cross-hairs in the center of the alignment markers.

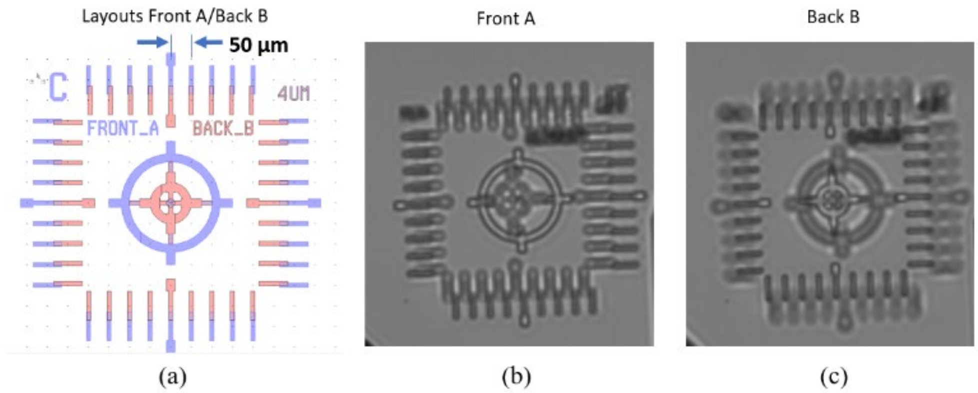

Figure S6 Front-back side misalignment inspection, (a) designed layouts, (b) micrograph focused on Front A side and (c) micrograph focused on B side.

Figure S7 shows misalignment measurements at different locations over individual wafers from designs V4, V5 and V6. For the V4 wafer #8, we can see the central alignment marker is very well aligned with an alignment error of less than ±3 µm in X and Y and the four outside markers located at 26 mm distance from wafer center showing an anti-clockwise angular rotation of around 0.045º. For designs V5 and V6 we do not have a central marker as the metalens itself is located in the wafer center. From the surrounding four alignment markers we can however estimate a shift ΔX = -15±3 µm, ΔY = 0±3 µm and Δθ = 0.044º clockwise for V5W15, and ΔX = -25±3 µm, ΔY = 0±3 µm and Δθ = 0.044º clockwise for V6. All results are summarized in Table S2.

Given the very similar values of Δθ found between the wafers, we hypothesize that this rotational misalignment could be systematic either in the tool calibration or in the notch shape when mirrored as the wafer flips over. Further evidence is provided by the difference in the counterclockwise rotational shift Δθ for V4 versus clockwise shift for V5 and V6. We traced this difference back to the orientation of the wafer, as each wafer has been provided by a laser mark on one side of the notch by the supplier. The laser mark on the notch was on the back side for the V4 as compared to the other two batches where the laser mark was on the front

side. Thus, the misalignment appears to be related to the wafer batch and may be calibrated out by careful characterization as is shown below.

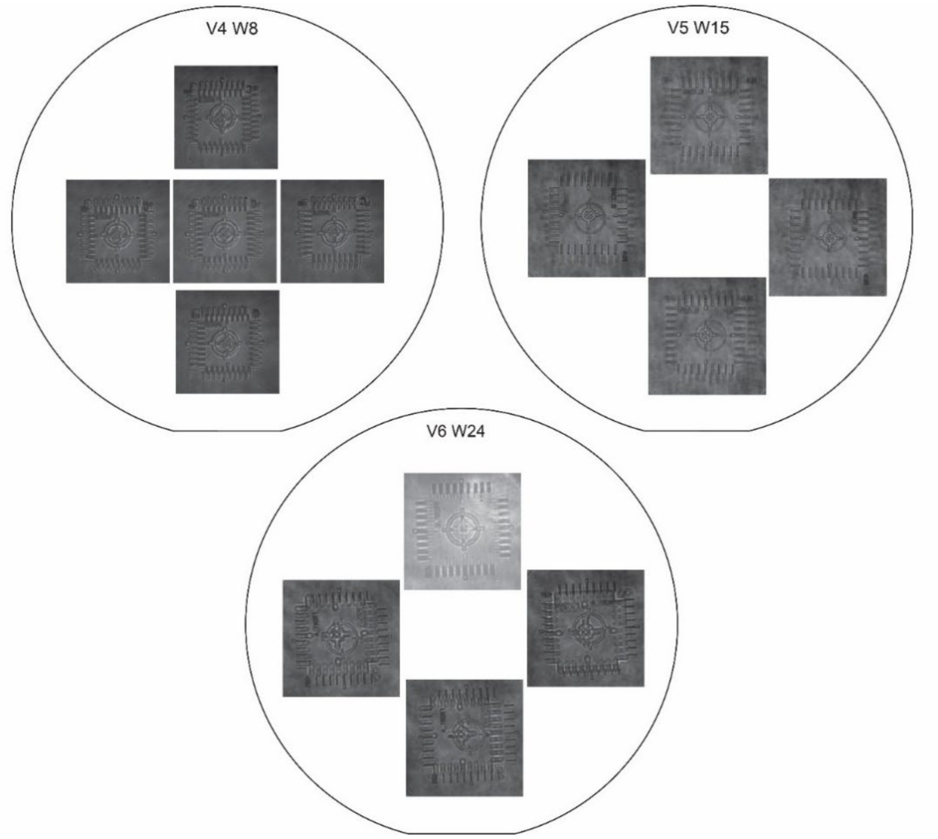

Figure S7 Front-back side misalignment inspection for three wafers V4 Wafer #8, V5 Wafer #15 and V6 Wafer #24.

To investigate whether indeed a better alignment can be achieved through a rotation offset, we applied a stage correction in both clockwise and counterclockwise directions to two wafers in the V5 batch. We developed two wafers with opposing wafer rotations of 0.045 degrees in the second exposure. Figure S8 shows results for wafers without correction (#15), and with rotational corrections for +0.045° (#16) and -0.045° (#17). We tested the rotational correction in both directions, in order to avoid any ambiguity in the direction of the rotation correction. Selected markers are in four quadrants corresponding to top right, bottom right, bottom left and top left. We note that these are different marker locations than those shown in Figure S7 which were closer to the horizontal and vertical axes.

Figure S7 clearly shows that for wafer #16, the misalignment is larger than for the uncorrected wafer #15, indicating that in this case the correction was applied in the wrong direction. For wafer #17 the correction has resulted in a reduced error in misalignment as

compared to the uncorrected wafer #15, where the residual misalignment remains <10 µm for each of the markers. We note that it is difficult to assign this residual misalignment to a specific shift or rotation, and some of this may be associated with the multi-exposure stitching involving and additional 90º wafer rotation as outlined in Figure S2.

From this study we conclude that it is possible to correct for systematic misalignment in the system, once the error has been identified, to below <10 µm residual error. Using the laser marked notch to identify a front side, we can keep track of the orientation of all wafers going through the system. So far, we have used one type of wafers from one supplier, and we cannot exclude batch variations. Therefore, it will be important to keep track of alignment accuracy going forward to establish a full understanding of this parameter between fabrication runs, when changing wafer batch, or even after DUV tool servicing.

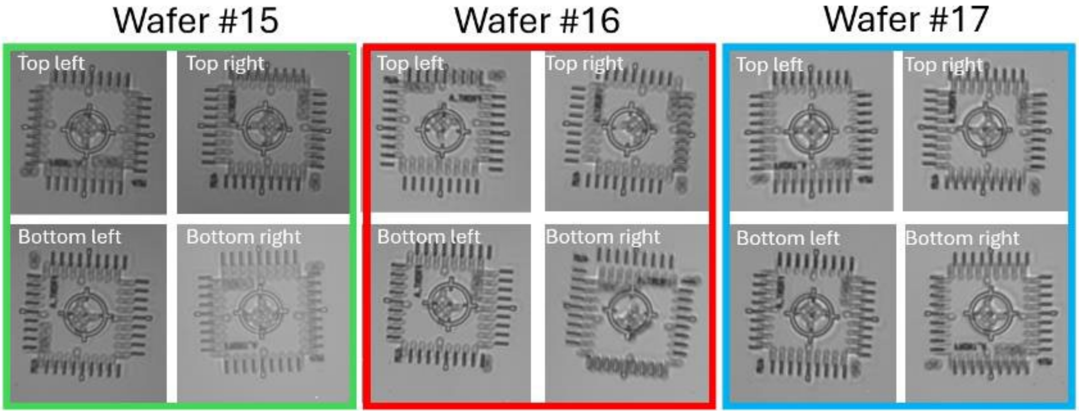

Figure S8 Wafer front-back side misalignment inspection for three different wafers V5 without correction (#15) and with rotation corrections of +0.045° (#16) and -0.045° (#17).

Table S2 Misalignment for different wafers.

Design	Wafer	ΔX (µm)	ΔY (µm)	Δθ °	Comment
V4	#8	0 ± 3	0 ± 3	-0.045 ± 0.005	Wafer front/back side reversed
V5	#15	-15 ± 3	0 ± 3	0.044 ± 0.005	
	#16	6 ± 3	0 ± 3	0.090 ± 0.005	Rotational correction +0.045°
	#17	-10 ± 3	0 ± 3	<0.01 ± 0.005	Rotational correction -0.045°
V6	#24	-25 ± 3	0 ± 3	0.044 ± 0.005	

S5. Large-area double-sided metalens V5 and V6 – design and characterization

To compare the results for front and back side for the probability of selection for each element in the design database, results for both sides are shown in Figure S9. Both sides have similar distributions, indicating that the simulation model has converged to a similar choice of elements on each side.

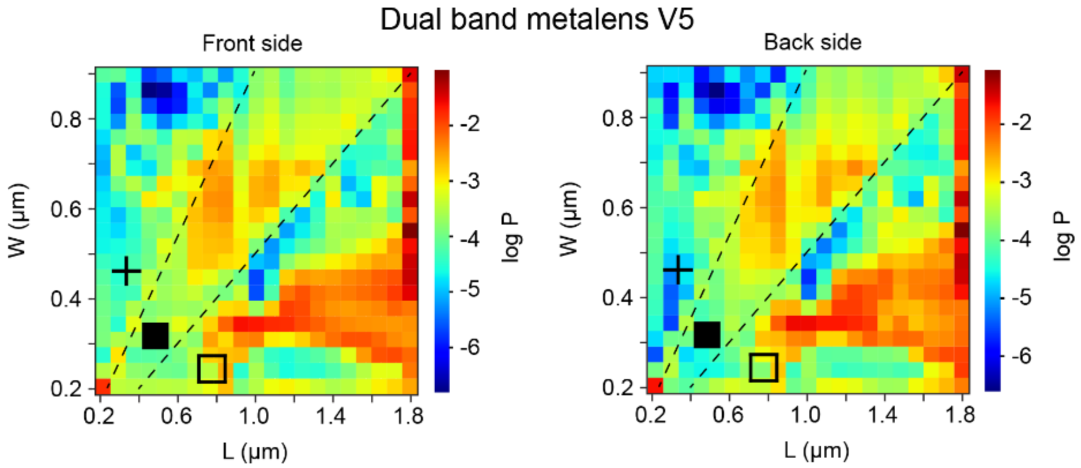

Figure S9 Probability of selection of individual elements in the design space of the large-area 40 mm diameter, dual-band metalens V5, for front side and back side.

For the single-band LWIR metalens design V6, the absence of constraints for MWIR performance allows for a revisiting of the design space as presented in Figure S10. We tested different periods and found that increasing the period to 2.6 μm resulted in a slight improvement in performance compared to the 2.2 μm period used in the dual-band design. Otherwise, a similar database model was chosen, consisting of hollow squares, filled squares and crosses, where the length and width parameters were varied from 0.2 – 2.3 μm and 0.2 – 1.0 μm, respectively. Following slight improvements in the experimental process optimization in the previous fabrication runs, we increased the achievable etch depth to 4.0 μm, which allows achieving a larger range of optical phase values. Figure S10b and S10b show the transmission and phase of the database for a single metasurface.

Using this database, we design the LWIR double-sided metalens V6 in MetaOptics Designer, using the same parameters as the dual-band but with only the single-wavelength optimization target. Resulting probability maps for the front and back side are shown in Figures S10c and S10d which shows that the model convergence on a number of well-defined bands within the 2D parameter space.

The simulated designs for the large-area double-sided metalens demonstrators are summarized in Table S3, showing the calculated AFE values in both bands. An efficiency increase up to 69.6% at LWIR is obtained by using the single-band design compared to the dual-band metalens with design performance at 49.9% AFE.

Table S3 Design parameters for the large-area double-sided metalens demonstrators.

Wavelengths (µm)	Focal length @4 µm (mm)	Focal length @10 µm (mm)	Height (µm)	Diameter (mm)	$\eta_{4\mu m}$	$\eta_{10\mu m}$
4/10 (V5)	80	80	3.5	40	28.4%	49.6%
10 (V6)	-	80	4.0	40	-	69.6%

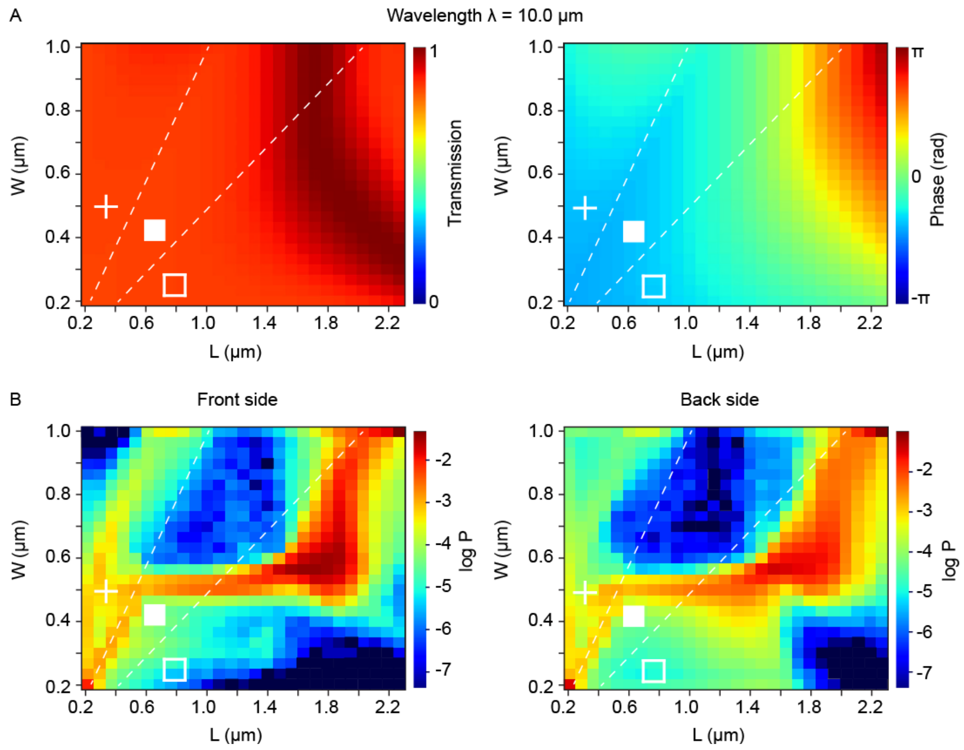

Figure S10 Design space of double-sided LWIR metalens. A Transmission and Phase of the database at design wavelength of 10 µm. B Probability of selection of individual elements in the design space of the large-area 40 mm diameter, dual-band metalens V6, for front side and back side.

S6. High-pass image de-hazing filter

The process of image enhancement by applying a high-pass enhancement filter is explained in Figure S11. The filter removes the characteristic haze of the metalens image below 3 lines / mm and produces a better contrast with markedly more visible background features. The process is implemented using Python as illustrated in Figures S11a and S11c. First, a high-pass filtered image is generated by subtracting a 30-pixel Gaussian low-pass filter from the original image, resulting in the high-pass image as shown in Figure S11b containing both negative and positive contributions. The high-pass enhanced image is shown in Figure S11b and is obtained by adding the original image to the high-pass image. The processing ensures that the original image is retained in the case of a flat background (no high-pass components) whereas features with spatial frequencies higher than 3 lines / mm are sharpened.

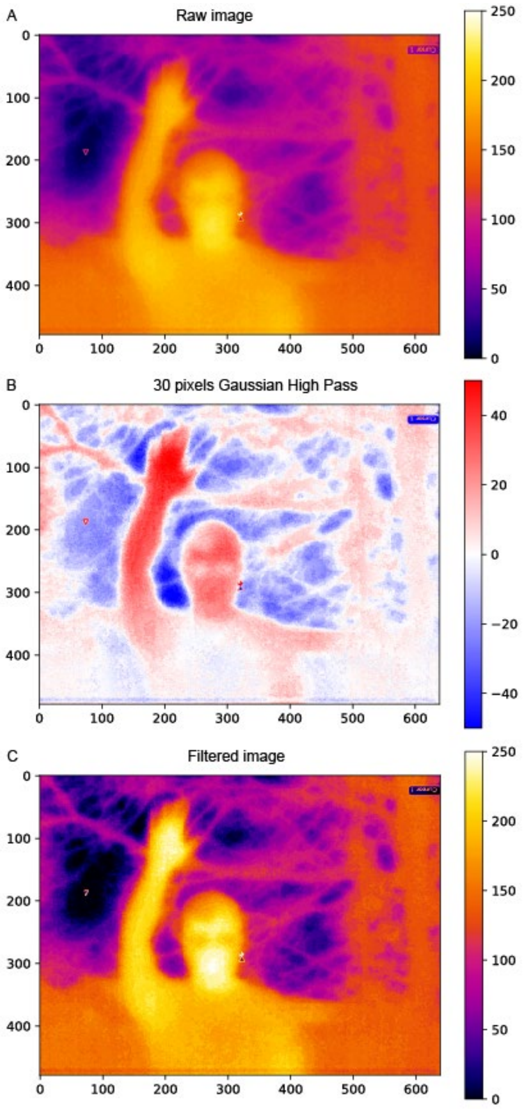

Figure S11 Details of image processing using a 30 pixels Gaussian filtering to reduce blurring and haze below 3 lines/mm spatial frequency.

S7. Dual-band double-sided metalens V4 – design and characterization

The details of the three dual-band double-sided metalens designs, for demonstrating independent control over focal length, are shown in Table S4. All lenses are 12.5 mm in diameter and with a design etch depth of 3.5 µm.

Table S4 Three dual-band metalens designs with different focal lengths at 4 µm and 10 µm wavelength.

Wavelengths (µm)	Focal length @4µm (mm)	Focal length @10µm (mm)	Height (µm)	Diameter (mm)	$\eta_{4\mu m}$	$\eta_{10\mu m}$
4/10	25	20	3.5	12.5	30%	40%
4/10	25	25	3.5	12.5	35%	48%
4/10	25	30	3.5	12.5	35%	50%

The peak efficiencies are extracted from the simulation results which are presented for the three different lenses in Figures S12 – S14. These results correspond to those presented in the main text Figure 3, but here with the full on-axis intensity maps shown for each lens separately with the color scale indicating the relative peak intensity as percentage of the incident power per µm^2. Integration of the intensity profile over the spot area yields the absolute focus efficiencies as given in panels c and d for each of the figures. Increasing the spot diameter (Area of Interest, AOI) from 8.8 to 52.8 µm gives an indication of how the intensity is distributed. The AOI sizes are given in multiples from 4x – 24x the simulation model step size of 2.2 µm (this is equal to the unit cell period).

Details of the fabrication of metalens design V4 are given in Figure S15, showing the two DUV reticles of front and back side (a), photograph of the Nikon DUV lithography system and Track used for the fabrication (b), final produced wafer front and back side (c) and detailed photograph of back side (d).

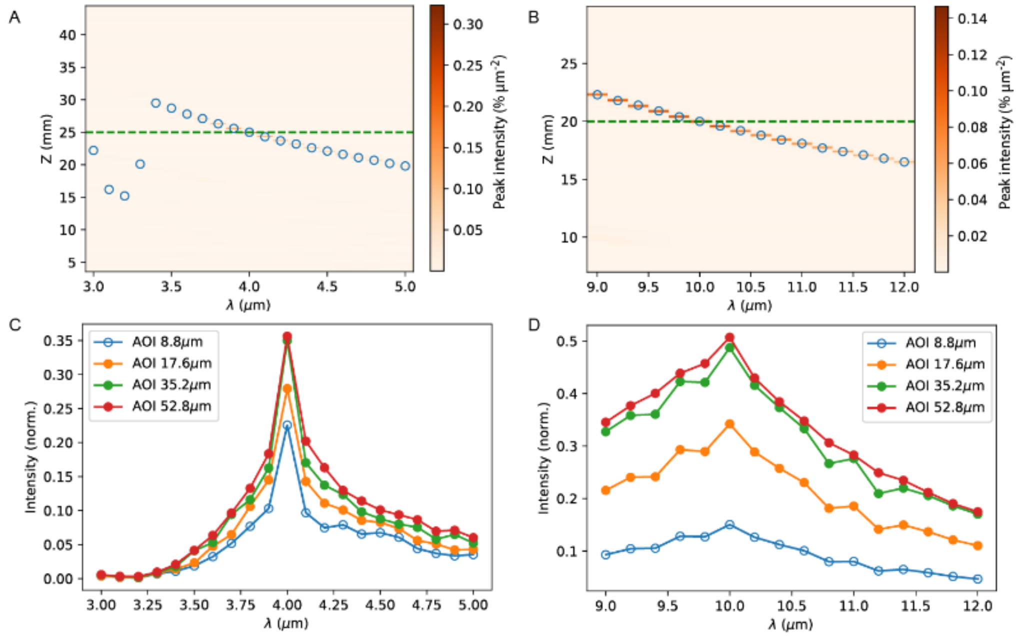

Figure S12 (a,b) Simulated on-axis intensity profiles (color map) with maximum intensity (open circles) for MWIR and LWIR for the lens design with f4µm = 25 mm and f10µm = 20 mm. (c,d) peak efficiency (total intensity normalized to incident) within a circular area of interest (AOI) from 8.8 – 52.8 µm diameter, for MWIR and LWIR.

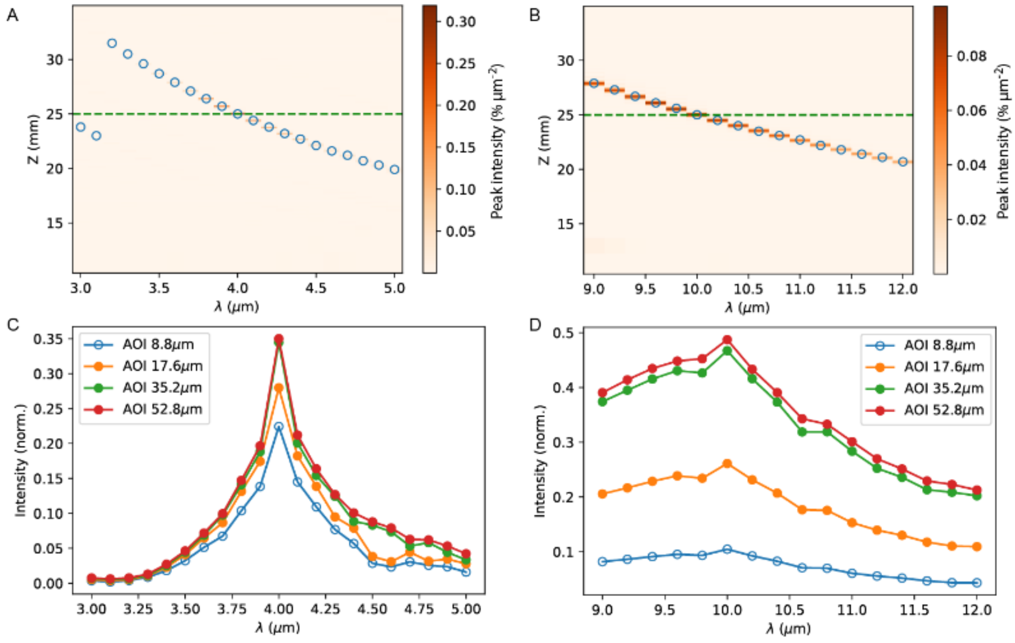

Figure S13 (a,b) Simulated on-axis intensity profiles (color map) with maximum intensity (open circles) for MWIR and LWIR for the lens design with $f_{4\mu m}$ = 25 mm and $f_{10\mu m}$ = 25 mm. (c,d) peak efficiency (total intensity normalized to incident) within a circular area of interest (AOI) from 8.8 – 52.8 µm diameter, for MWIR and LWIR.

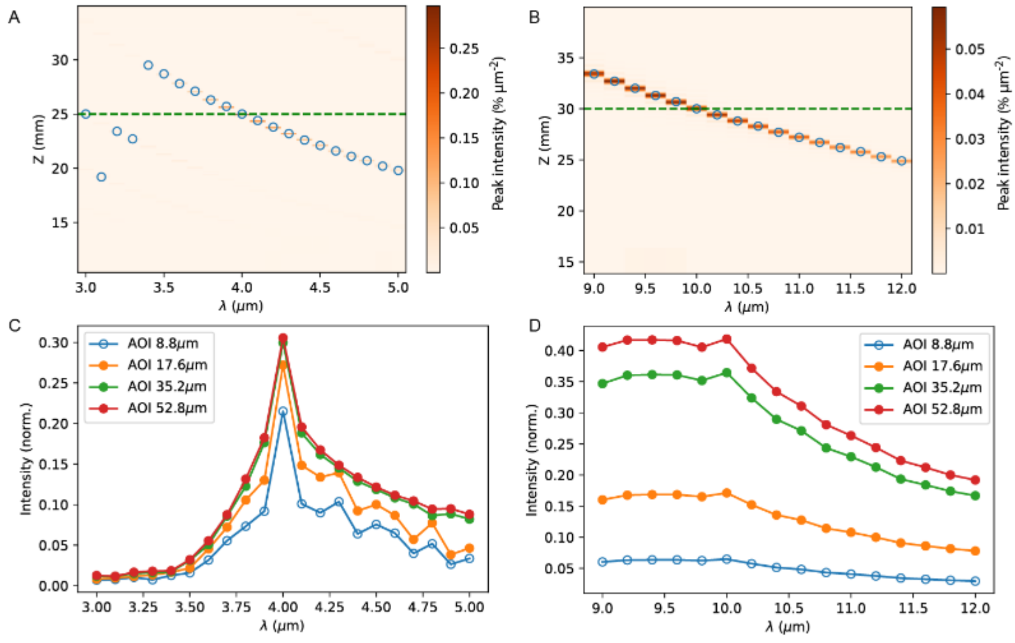

Figure S14 (a,b) Simulated on-axis intensity profiles (color map) with maximum intensity (open circles) for MWIR and LWIR for the lens design with f4µm = 25 mm and f10µm = 30 mm. (c,d) peak efficiency (total intensity normalized to incident intensity) within a circular area of interest (AOI) from 8.8 – 52.8 µm diameter, for MWIR and LWIR.

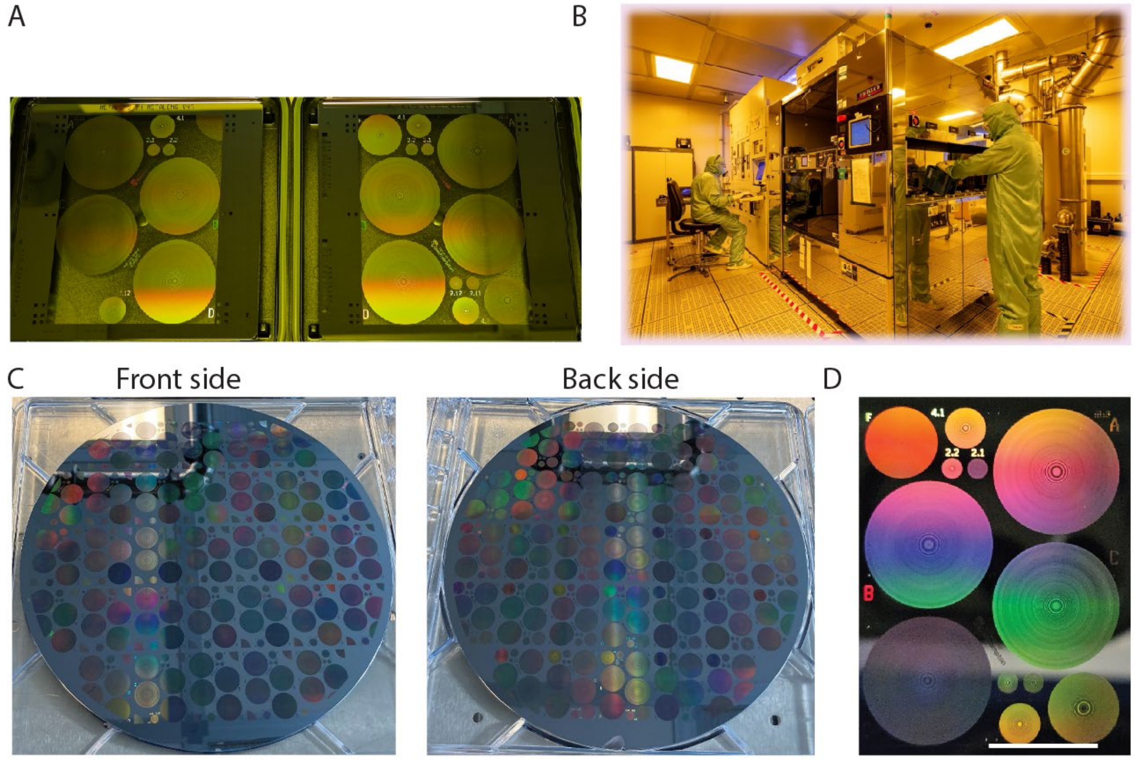

Figure S15 (a) pair of DUV reticles for front side and back side of metalens wafer V4. (b) Photograph of DUV Scanner and Track used for the double-sided DUV lithography fabrication. (c) Fabricated wafer with 12.5 mm diameter double-sided metaoptics, showing front side and back side. (d) Zoomed-in optical photograph showing single write field with various metalenses, lenses labelled A, B, C are used in this study.

A full set of optical measurements results from wafer V4 are shown in Figure S16. The experimental setups are schematically shown in Figure S16a, for configurations where the 2.1×1.9 mm^2 MEMS blackbody light source is imaged directly using the metalens onto the mid-wave or long-wave camera (top / bottom setups), or where the blackbody source is used to illuminate a 1951 USAF test target (middle setup). We were unable to apply the USAF target imaging with the LWIR arrangement due to the presence of a SiO_2 substrate preventing transmission of blackbody in this region. The matching imaging lenses are Janos Nyctea 50mm for the MWIR camera and FLIR T197922 imaging lens for the LWIR camera. All images at different spectral band filters are presented in Figures S16b-d for the three types of metalenses. Figure S16c shows the detail of the blackbody light source where the bottom right corner was imaged on MWIR and the full area on LWIR, the difference in field of view being caused by the different imaging optics in the two setups.

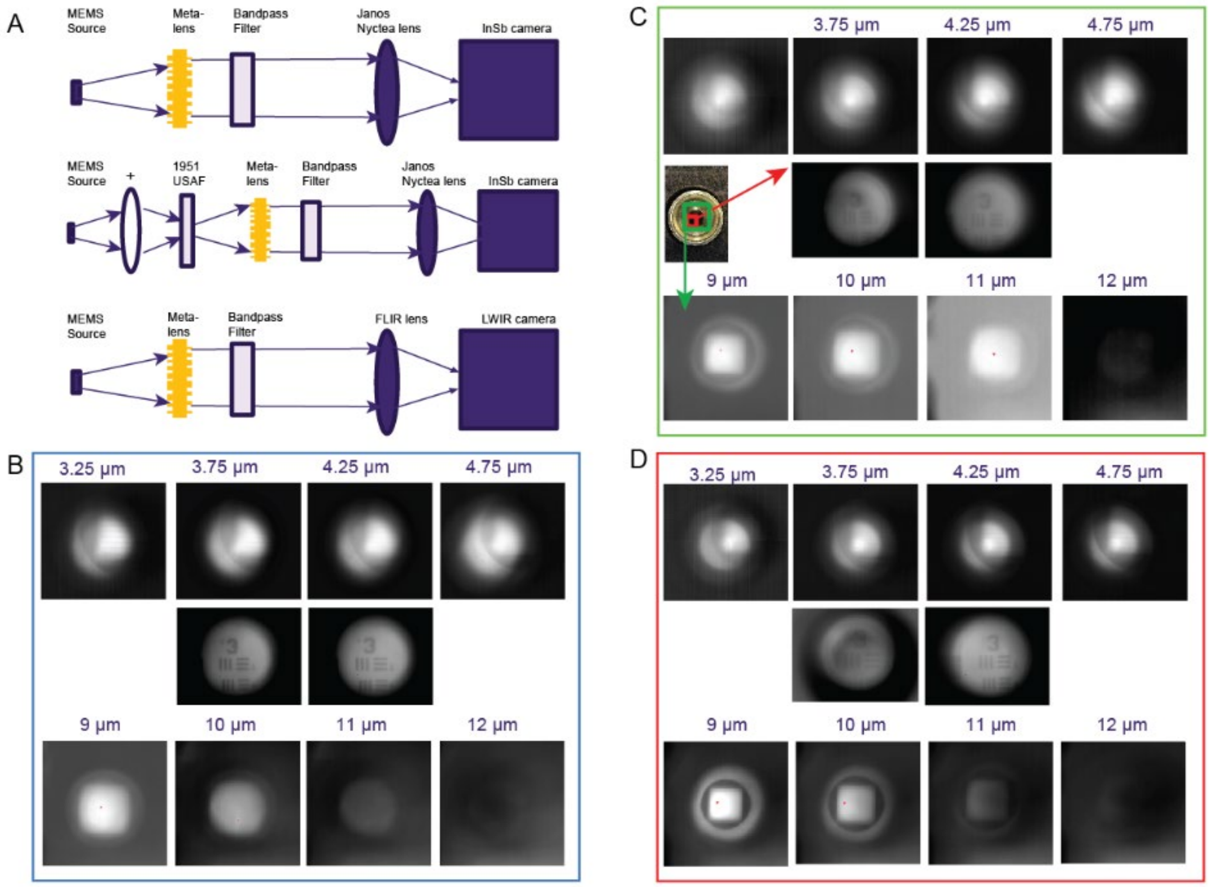

Figure S16 (a) Schematic illustration of three different setups for measurement of the focal length using direct imaging of blackbody light source or of a 1951 USAF resolution target. (b-d) Full set of images obtained by direct imaging through a series of spectral bandpass filters in MWIR and LWIR range, for the three different metalenses of Table S2. Measurements of 1951 USAF target could only be obtained for wavelengths of 3.75 μm and 4.25 μm.

Absolute focus efficiency (AFE) for the metalens V4 was obtained using a CO_2 laser at 10.8 μm wavelength. Figure S17a shows the experimental arrangement, where a 200 μm pinhole was used to filter the focused light from unfocused background. The focus was imaged onto the LWIR camera using a set of neutral density filters to strongly attenuate beam in order to prevent damage to the uncooled sensor. Focal spots are shown in Figure S17b for the three different metalenses showing a focus of around 2-3 pixels on the camera. Using the same arrangement, the pinhole was placed in the focal plane and aligned to transmit the laser spot. After alignment, an optical power meter was placed behind the pinhole to measure the transmitted power. This power was normalized to the incident power without pinhole and metalens to obtain the AFE as shown in Figure S17c. AFE values of around 25% were obtained experimentally, compared to simulated values of between 30%-35% at the wavelength, indicating a performance of the metalens which is in reasonable agreement with the design.

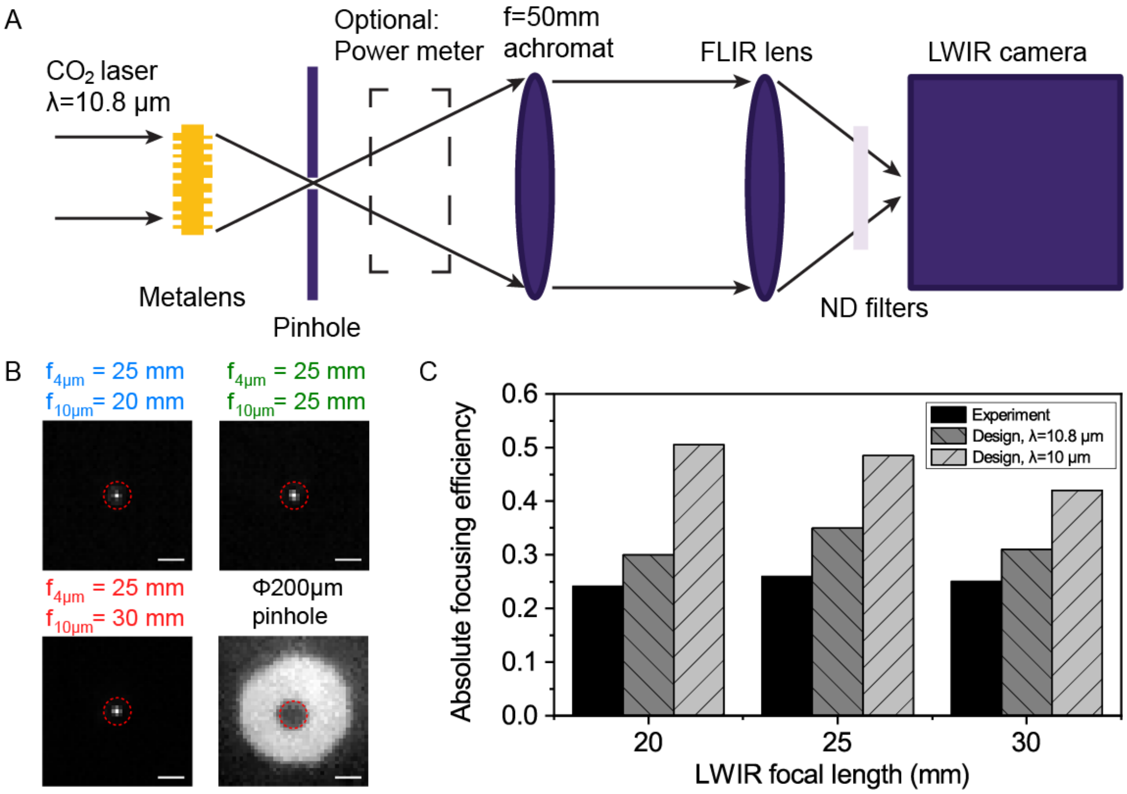

Figure S17 (a) Schematic of setup for measuring of absolute focusing efficiency using CO_2 laser at 10.8 μm wavelength. (b) Images obtained using LWIR camera of focus spot produced by metalens using achromatic collection optics, as well as of pinhole surface placed in focal plane of collection lens. Scale bars are 200 μm. (c) Absolute focusing efficiency experimentally determined using power meter placed after pinhole, compared with simulated design performance at 10.8 μm and 10 μm wavelength (maximum of design).

S8. Imaging of University of Southampton logo test sample

Details of the University logo test sample are shown in Figure S18. The logo test sample is designed as a three-layer stack (Figure S18a) using a Transformation Matrix method with the peak emissivity (absorption) at 4 μm. The stack consists of 80 nm Al:ZnO, 1000 nm SiO_2 and 100 nm Al as a back reflector. The Al:ZnO (AZO) was grown using atomic layer deposition with TMA, DEZ and DI water as precursor and a TMA:DEZ cycle ratio of 1:24, at a temperature of 250 °C. The SiO_2 and Al were grown using a Helios sputtering system. Detailed process information is available in our previous publication, Sun *et al.* Adv. Mater. 2020, 2001534. The infrared response is measured by an FTIR system, resulting in the emissivity spectra presented in Figure S18c with the measurement locations detailed in Figure S18b.

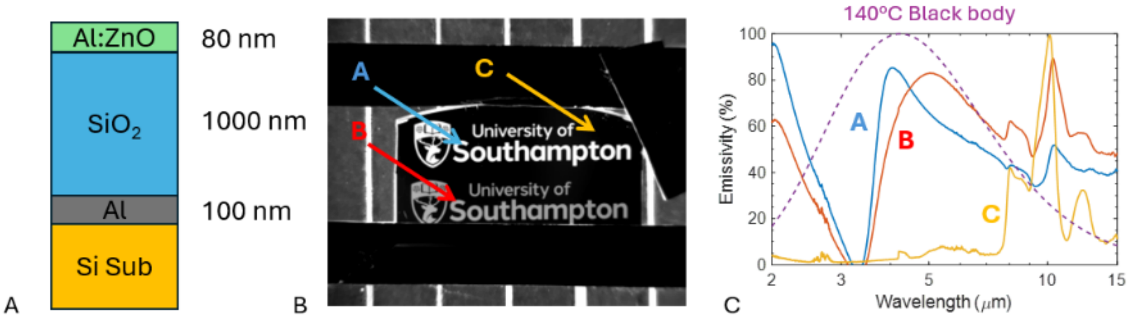

Figure S18 University logo structures and infrared response. (a) three-layer stack with high conductive Al:ZnO on SiO_2 on Al, (b) Thermal imaging with locations labelled, (c) Emissivity spectra measured at labelled locations, with black body spectra presented at 140 °C.

Figure S19 shows the experimental setup for imaging of a heated test object, for the long-wave (a) and mid-wave (b) range. The infrared camera is positioned at approximately 42 cm distance from the test sample. For the long-wave setup, the hybrid optical stack consists of a 25 mm diameter BaF_2 refractive lens and 40 mm diameter metalens which are independently mounted. The BaF_2 optic is retained in a 2-inch diameter lens tube and the metalens in a 42mm diameter T-mount lens tube. The two tubes slide into each other to provide a compact tunable optical system with relative positions controlled by using two manual translation stages. The BaF_2 optic is positioned very close to the LWIR camera at an estimated distance of around 15mm from the focal plane array, this was possible due to the uncooled camera offering good access close to the focal plane array. For the mid-wave setup, the cooled InSb camera CEDIP/FLIR SC7300 Titanium has a cold chamber with limited access, therefore the hybrid refractive and metasurface configuration could not be used. In this case, the metalens was used by itself in a singlet configuration.

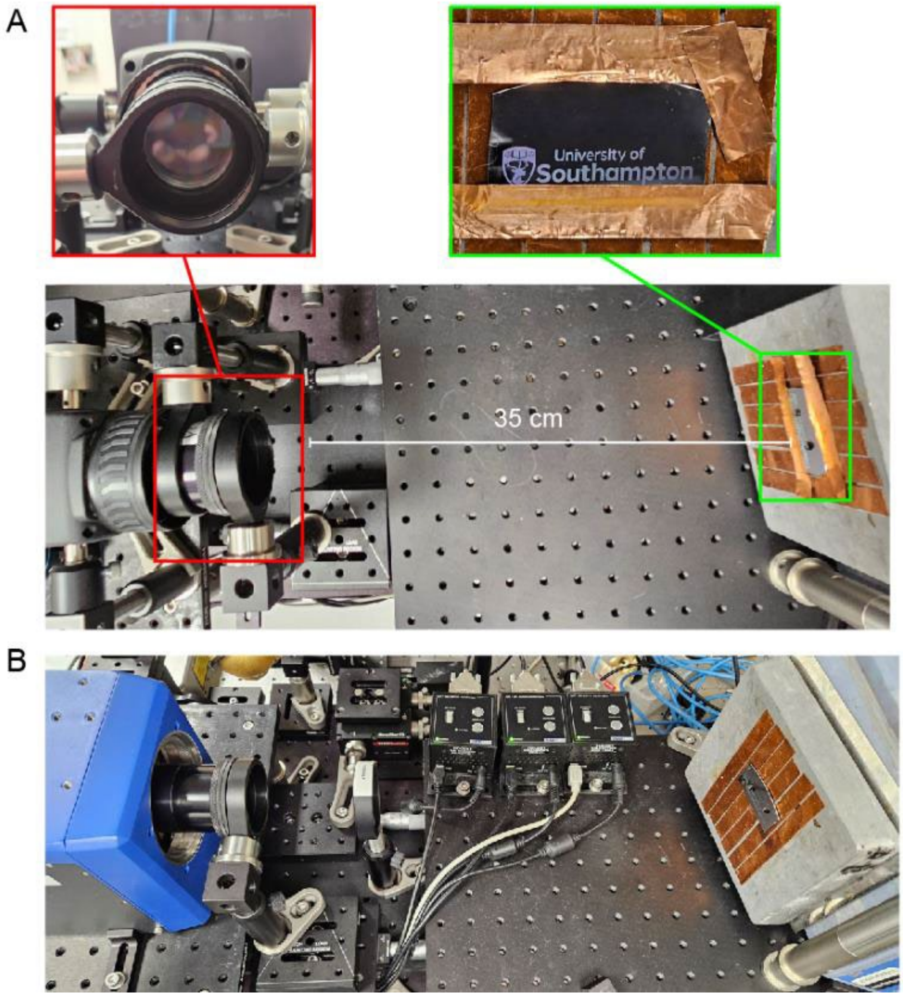

Figure S19 Experimental setup for imaging of Logo test target at 140 ºC. (a) Long-wave imaging setup using FLIR A655sc LWIR camera, hybrid optical system consisting of BaF_2 refractive lens and metalens (front of metalens shown in inset) and vertically mounted hot plate with sample attached using copper tape. (b) Setup for Mid-wave imaging using CEDIP/FLIR Titanium camera with metalens (no BaF_2 refractive lens).

S9. Hybrid metaoptic and refractive compound lens

To put into perspective the performance of the hybrid metalens – refractive BaF_2 configuration, we performed the same LWIR imaging of the target object using just the metalens without refractive optic. Figures S20a and S20b show results for both the dual-band V5 (a) and LWIR-only V6 (b) metalenses. For both metalenses, the images are unsharp and with very low contrast on the university logo. The outlines of the copper tapes are also much less clear and the lines of emission between the adjacent copper strips are hazy. We attribute this difference to the lack of chromatic correction for the metalenses over the bandwidth, thus showing the good performance in comparison by the hybrid metalens – refractive optical system of which results are presented in the main text.

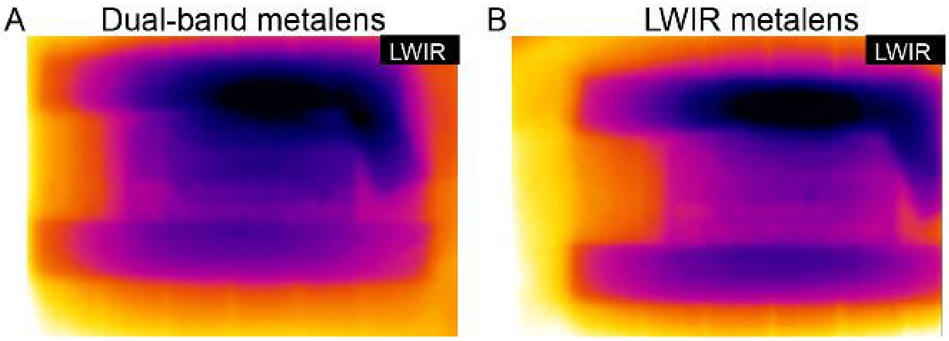

Figure S20 Images of target object heated to 140° C for singlet metalenses without the BaF_2 hybrid arrangement, for (a) dual-band metalens (V5) and (b) LWIR metalens (V6).

S10. Additional imaging of dual-band metalens

We have tested the fabricated 40 mm dual-band metalens V5 in real-life imaging with MWIR/LWIR cameras. MWIR imaging was performed for a heated soldering iron at 150°C with the black-body emission in the MWIR band and presented in Figure S21a comparing with the camera's original Nyctea imaging lens Figure S21b. The Metalens imaging shows clearly the soldering iron details. Compared with the original Nyctea lens imaging, the metalens image is a bit blurry. This is attributed to the chromatic aberration of metalens.

LWIR imaging was performed for a human hand with images shown in Figure S22 for the 40 mm dual-band metalens (a), commercial CaF_2 lens (b) and commercial BaF_2 lens (c), both refractive lenses are 25mm in diameter with 50mm focal length and f-number F/2. All three singlet lenses have strong chromatic aberrations individually, with the CaF_2 having a higher dispersion than the BaF_2 lens. To achieve improved chromatic aberration control, we adopted the developed compound system with one metalens and one CaF_2 or BaF_2 lens for aberration correction, with resulting images are presented in Figure S22. The metalens/CaF_2 hybrid (Figure S22a) gives a good quality image in LWIR band with an approximately 2 times larger field of view than the individual F/2 singlets. A similar improvement is also seen using the Metalens/BaF_2 hybrid combination (Figure S22b) which achieves a higher signal to noise due to the higher overall transmission of the BaF_2 lens. Figure S22c shows a LWIR image of the soldering iron at 150°C using the Metalens/BaF_2 hybrid. These real-world imaging demonstrates that the compound solution can offer an effective way to correct metalens aberration and provide good imaging.

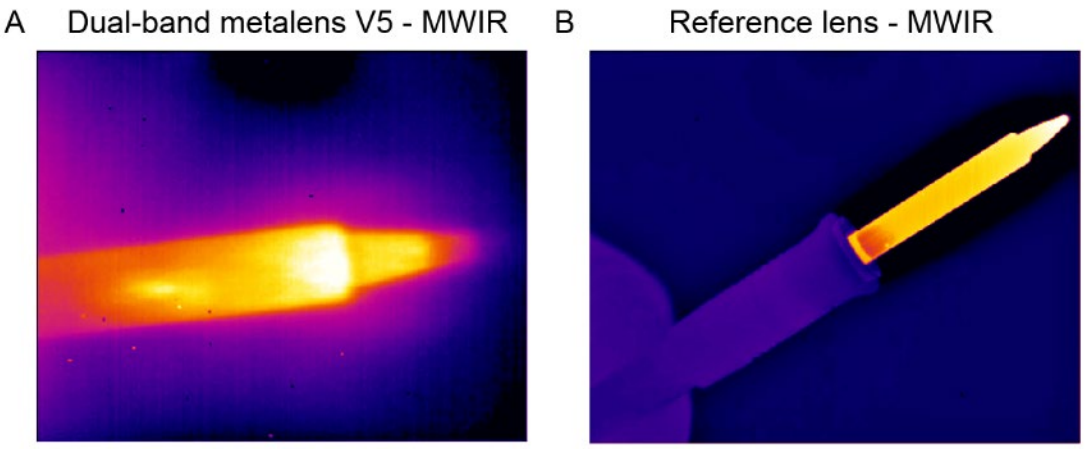

Figure S21 MWIR imaging measurement through InSb camera for a 150 °C soldering iron at a distance of 1 m from the camera, by dual-band metalens V5 (a) and the Nyctea 50 mm lens (b).

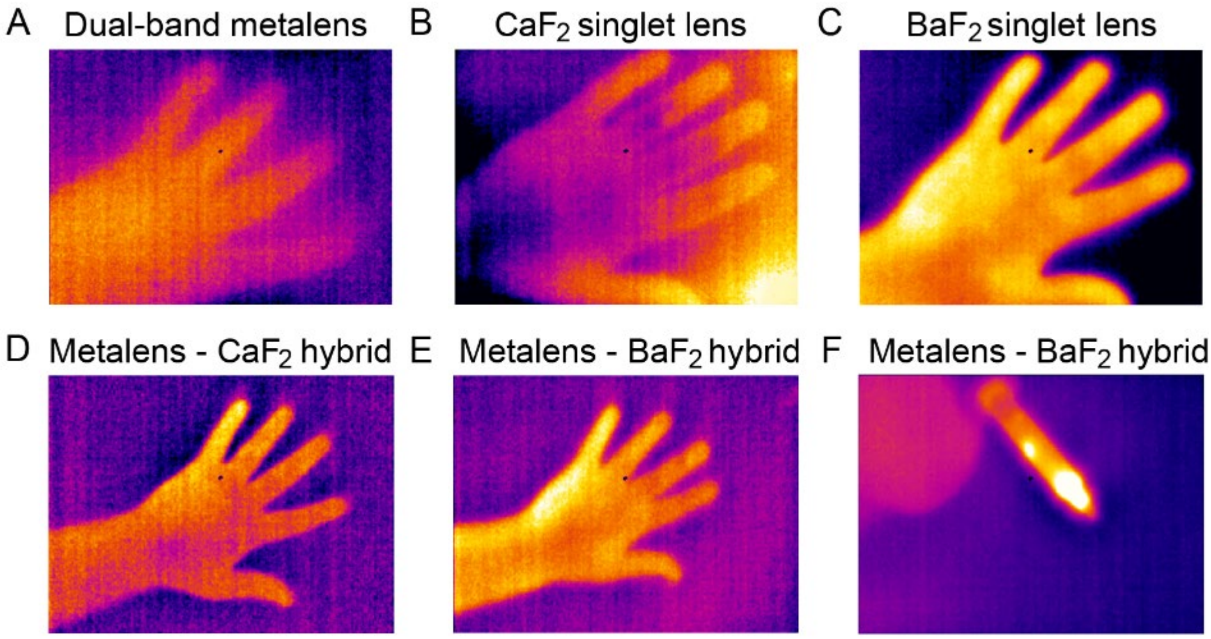

Figure S22 (a-c) LWIR imaging measurement using singlet lenses for dual band metalens (a), CaF_2 lens (b) and BaF_2 lens (b). (d-f) Compound system imaging using metalens - CaF_2 hybrid (d), metalens-BaF_2 hybrid. Hand in (a-e) was kept in same position and distance for all images. (f) LWIR image of soldering iron at 150 °C using metalens-BaF_2 hybrid.

S11. Outdoor LWIR demonstrator

For the outdoor demonstrator the LWIR camera system was set up pointing towards a scene with objects at distances from 10 – 150 m as illustrated in Figures S23a and S23b . Figure S23a illustrates the local topography of the Highfield campus at the University of Southampton, where the camera is located in the corner of building 46 and pointing in the direction of building 40. The roof of building 40, located at around 150 m distance, is clearly

seen in the optical photograph of Figure S23b showing the elevation of this structure. As the optical image is taken at a slightly higher vantage point than the infrared demonstration, the alignment of the person with the roof is not present here, as this is seen more clearly in the infrared images which are taken from the height of an optical bench. The infrared images are optimized to maximize the visibility of the branches of the trees, located at around 25 m distance. Figures S23c-f shows the raw and processed images from the metalens – refractive BaF_2 hybrid optical system.

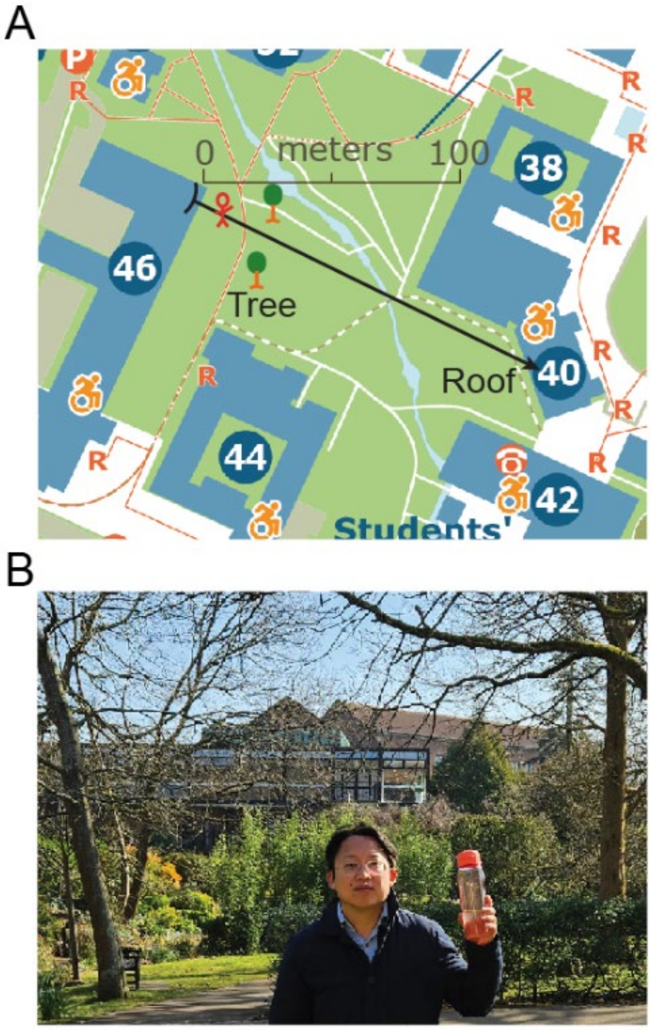

Figure S23 (a) Map detail showing geographic arrangement to scale with camera position, person at 10m, trees at 25 m, and rooftop at 150 m distance. (b) Visible-band photograph of scene used for outdoor demonstrator.

S13. Performance testing of large-area metalenses

Lens performance was evaluated by Teledyne Qioptiq Ltd using specialized setups commonly used in the optical system manufacturing industry.

LWIR analysis.

RMS. RMS quantifies the overall wavefront error, with 0.042λ indicating relatively smooth deviations compared to the wavelength. A low RMS value implies high optical quality, aligning with the Strehl ratio of 0.93 (see below). Interpretation: The optical system has minor residual imperfections.

Peak-Valley. PV measures the extreme deviations in the wavefront error. For the experimental data, 0.28λ shows slightly higher error than the simulation (0.2467λ). Experimental vs. Simulation: The slight difference suggests environmental factors, manufacturing errors, or alignment inaccuracies influencing the experimental data.

Strehl Ratio. A Strehl ratio of 0.93 indicates excellent optical performance (ideal Strehl ratio = 1 indicating that an optic is perfect and aberration free). At SR>0.8, the system is diffraction-limited, meaning its performance is governed by fundamental physics rather than imperfections. Interpretation: The wavefront quality allows for near-ideal focusing.

Astigmatism. This represents cylindrical distortions, with a magnitude of 0.06λ, oriented at -87.1°. This small value implies minimal elongation of the point spread function (PSF). Interpretation: Astigmatism is a minor contributor to the overall error.

Coma. Coma introduces asymmetric distortions in the PSF, typically caused by off-axis angles. A value of 0.27λ at 153° indicates moderate coma contribution.

Spherical. Spherical aberration is the largest contributor among measured aberrations, at 0.30λ. This results from the inherent curvature of optical surfaces, causing edge rays to focus differently from central rays. Interpretation: Spherical aberration limits the Strehl ratio slightly.

Experimental vs Simulation. The experimental PV (0.28λ) is close to the simulation PV (0.2467λ), indicating the system behaves predictably under real conditions. Discrepancy: Slight deviations may stem from environmental factors, material inhomogeneities, or manufacturing tolerances.

Summary. The low RMS (0.042) and high Strehl ratio (0.93) confirm excellent wavefront quality. Residual errors are dominated by spherical aberration (0.30), followed by coma (0.27), with minor contributions from astigmatism (0.06). Experimental results closely match simulation, validating the model's accuracy.

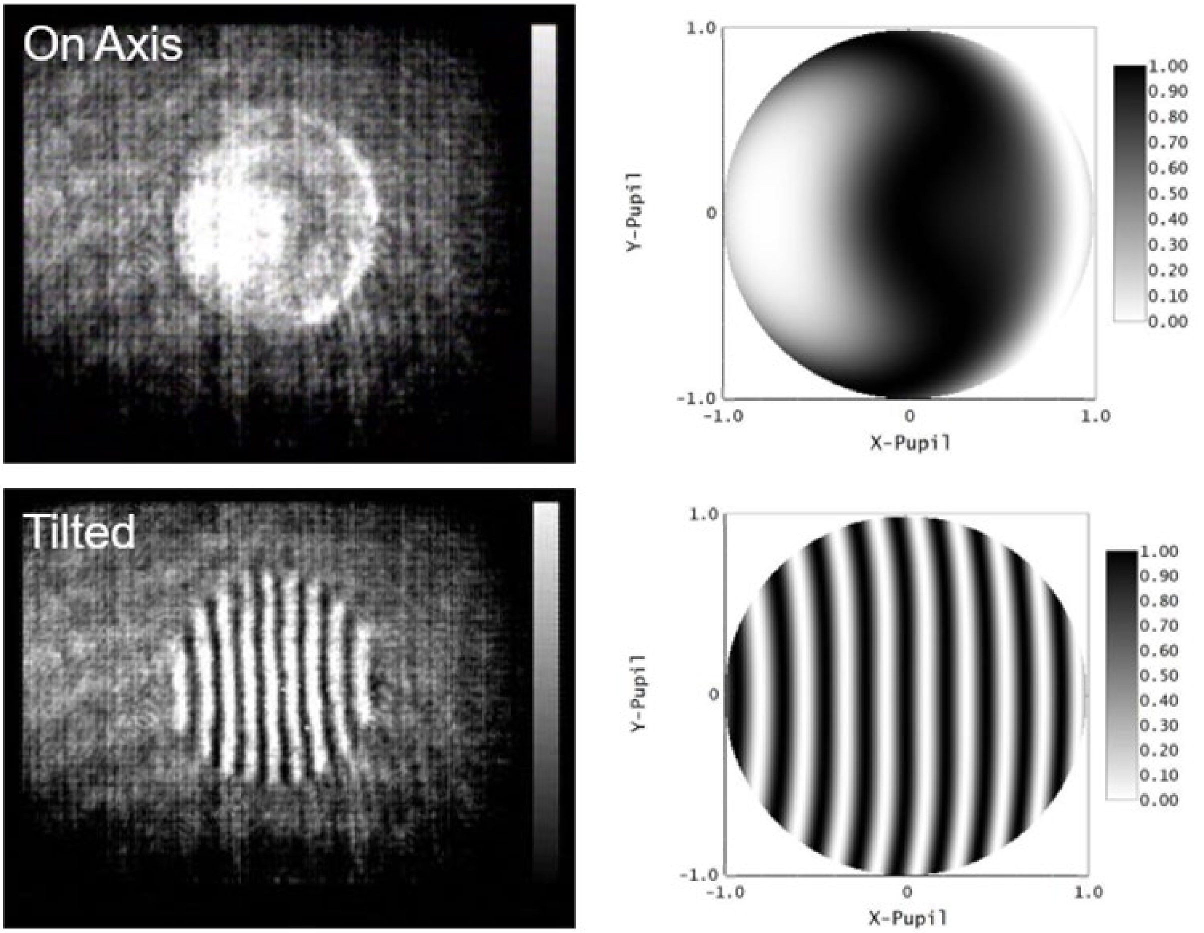

Figure S24 LWIR interferometry results at 9.24 μm wavelength, showing on-axis defocus and off-axis tilt, experimental (left) and simulations (right) for a peak-valley of 0.2467λ.

MWIR analysis.

The wavelength of 3.39 μm was dictated by the availability of a light source at this wavelength which is quite far from the design wavelength of the metalens. The measurements were done by removing / adding power at focus. Removing power at focus isolates higher-order aberrations (like astigmatism, coma, and spherical aberration) by eliminating the dominant curvature effects. This allows the analysis to focus on the residual wavefront errors without contributions from lens focusing power.

Purpose: Removing power at focus simplifies the evaluation of optical imperfections, as curvature can dominate the wavefront error and mask subtler aberrations.

Implication: This is particularly important in metrology setups where higher-order aberrations, not corrected by removing power, are the focus of analysis.

Deliberately introducing defocus, especially with power, allows for controlled testing of optical behavior under non-ideal conditions, which can simulate real-world scenarios or stress-test optical performance.
Purpose: To evaluate the robustness of the system's aberrations, Strehl ratio, and RMS wavefront error when subjected to misalignment or off-axis effects.
Implication: This configuration exaggerates contributions from tilt, astigmatism, coma, and spherical aberration, providing insights into how the lens performs when improperly aligned.

RMS. The RMS wavefront error quantifies the standard deviation of the optical wavefront deviations from an ideal flat wavefront. A value of 0.051λ is measured, which indicates minimal deviation, suggesting high optical quality. **Significance**: For high-performance systems, an RMS error below 0.07λ is typically considered diffraction-limited, where system performance is primarily governed by the diffraction of light rather than aberrations or imperfections.
Peak-Valley. PV = 0.239λ: The peak-valley metric represents the difference between the maximum and minimum wavefront deviations. A low PV value like this suggests minimal surface or optical imperfections. **Limitations**: While PV provides an overall sense of optical error, it is less robust than RMS because it can be dominated by localized defects.
Strehl Ratio. A Strehl ratio of 0.9024 confirming optical system is diffraction limited as it exceeds the threshold of 0.80.
Astigmatism. Astigmatism = 0.56λ (at -18.1°). This is a second-order aberration where the wavefront has different curvatures along perpendicular axes, causing lines in one orientation to focus differently than lines in another. Astigmatism of this magnitude indicates moderate asymmetry, likely due to lens imperfections or stress during mounting, or the purposely added tilt.
Coma. Coma = 1.02λ (at 5.9°). Coma is a third-order aberration caused by off-axis light rays forming comet-shaped blur spots. The relatively high value of coma indicates significant asymmetry, due to misalignment with the purposely added tilt.
Spherical. Spherical = 0.06.: Spherical aberration arises when marginal rays (closer to the edge of the lens) and paraxial rays (near the center) focus at different points. A value of 0.06 indicates a negligible contribution, suggesting high-quality lens design.
Summary. The low RMS and PV values, combined with high Strehl ratio, indicates the lens has good optical quality and is close to diffraction-limited performance.

Coma and astigmatism dominate the residual wavefront error, likely due to the purposely introduced tilt causing alignment issues.

Spherical aberration is negligible, suggesting the lens is well-corrected for this high-order aberration.

(a)

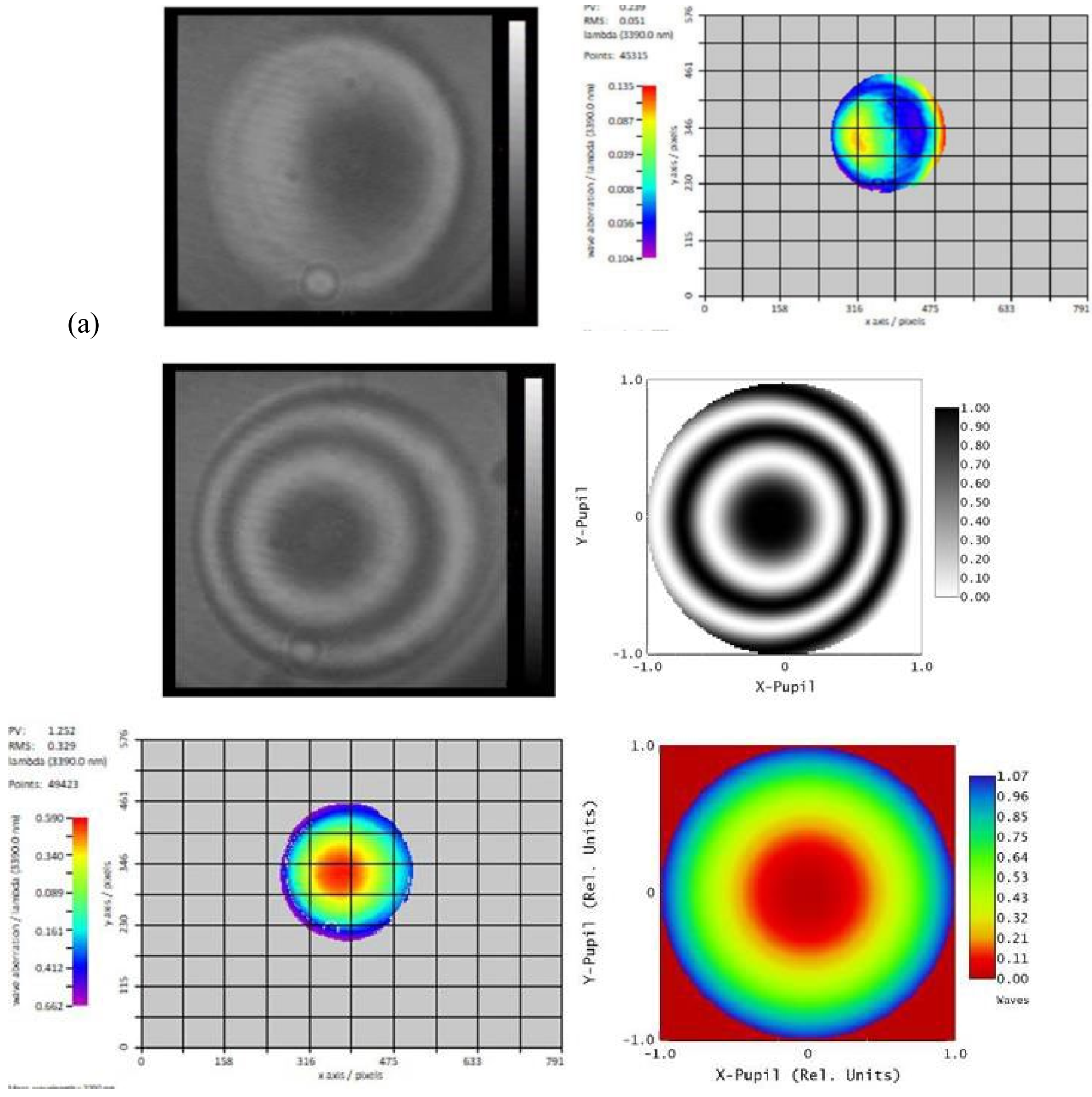

Figure S25 MWIR interferometry results at 3.39 μm wavelength, showing on-axis defocus and off-axis tilt, experimental (left) and simulations (right) for a peak-valley of 0.2467λ.

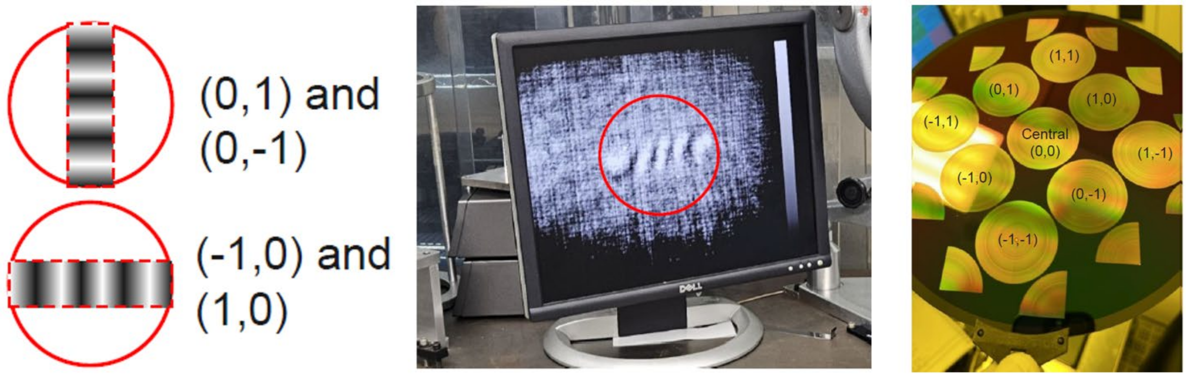

Figure S26 Variation of fringes seen in interferometric analysis for off-centre lenses, as labelled by coordinates on the wafer (right).

The central (0,0) lens on the wafer was found to be an excellent quality lens. The adjacent lenses displayed a misalignment on both sides as shown in Figure S26. As such, lenses positioned in (0,1), (0,-1), (1,0), and (-1,0) displayed a “letter box” style transmission window, although the wavefront transmitted appears subjectively good, it could not be quantified. Lenses positioned in (-1,1), (1,1), (-1,-1), (1,-1) had little to no transmission window for the interferometric analysis. This observation is consistent with the rotational misalignment between the two sides of the wafer, which results in very small misalignment in the center, and systematic shifts either horizontal or vertical in the off-center lenses.

S14. Wafer-scale testing of large-area metalenses

Variation of the metalens performance across the wafer was characterized for the dual-band metalens (V5) using an automated wafer positioning system. Direct imaging of a soldering iron was done by positioning the wafer in front of the camera as shown in Figures S27-S29. Both the MWIR and LWIR cameras could be used in this arrangement.

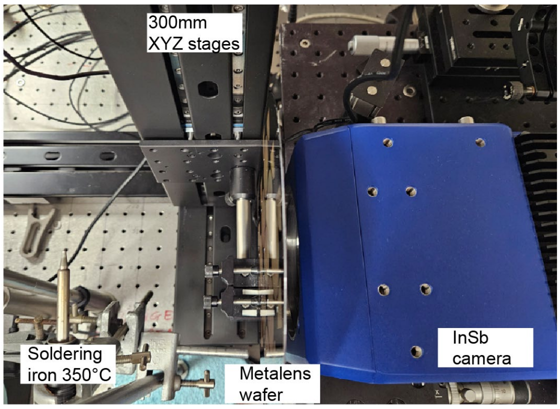

Figure S27 Optical setup for direct imaging with on-wafer metalenses to verify performance variations across the wafer, showing metalens wafer on 300 mm XYZ wafer positioning stage, soldering iron target and InSb camera.

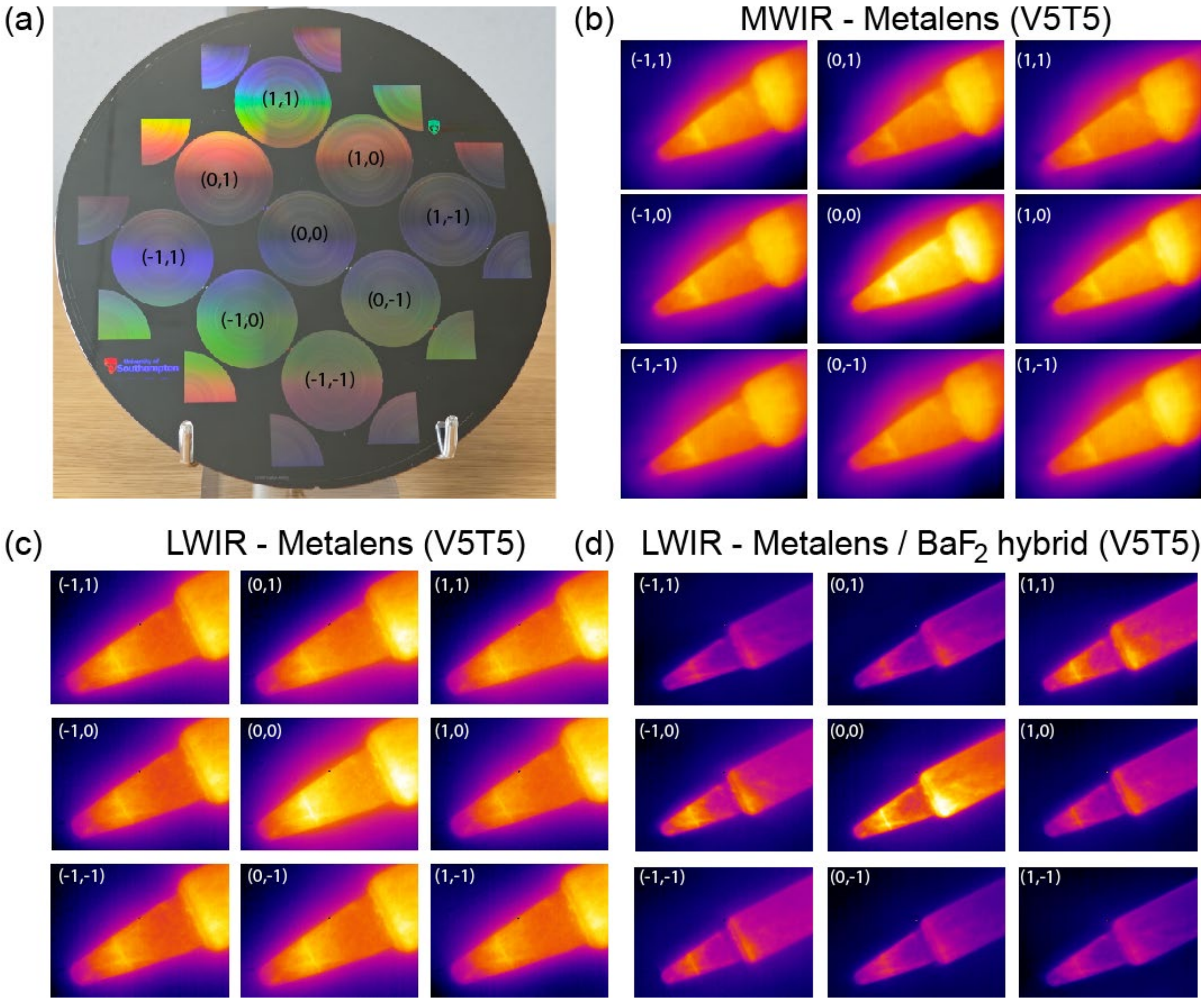

Figure S28 Wafer-scale characterization results for metalens imaging of wafer #T5. (a) Layout with labelling convention of metalenses on wafer. (b,c) Broadband MWIR and LWIR images (no filter) using metalens and (d) images using hybrid metalens – BaF_2 configuration.

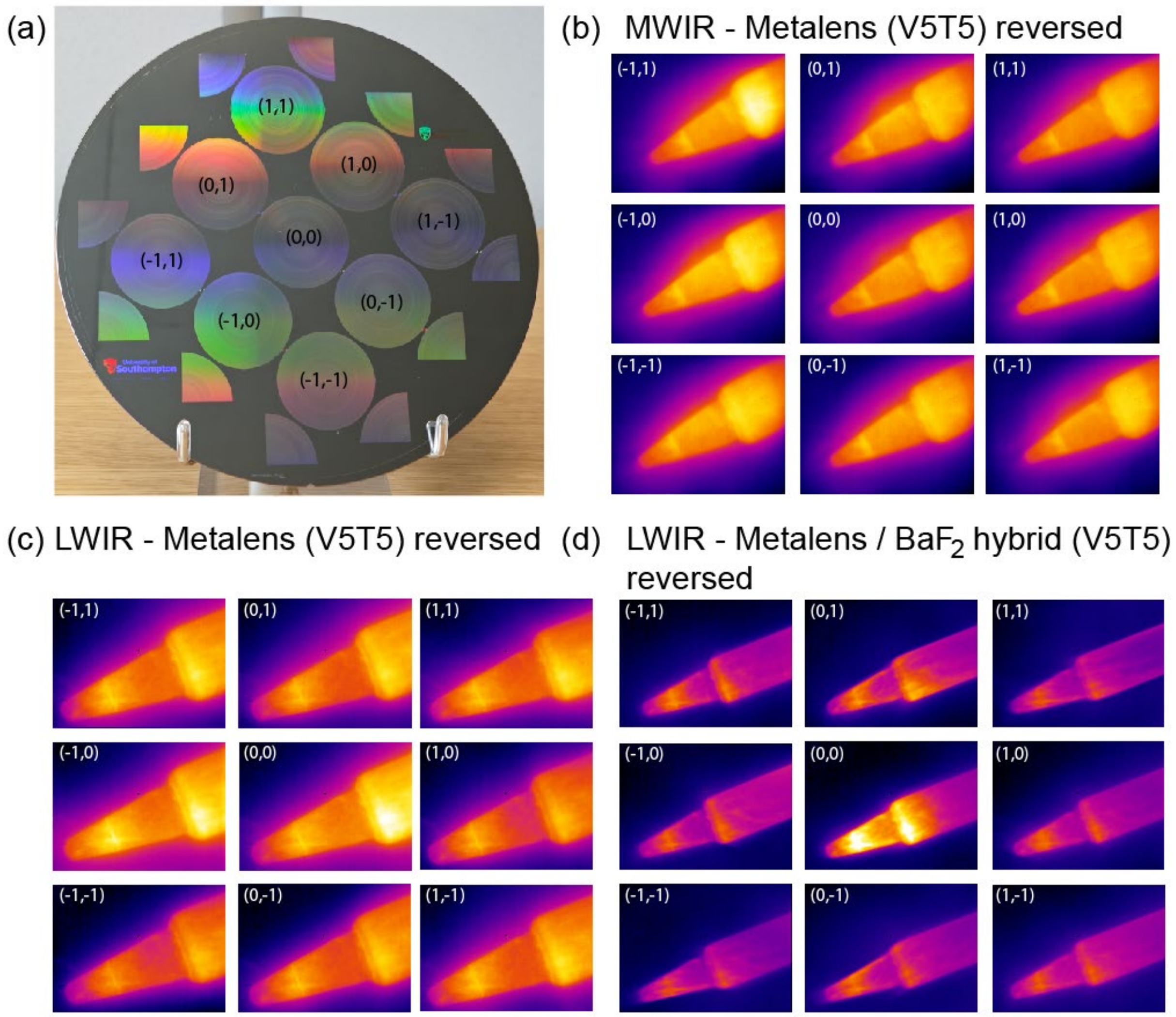

Figure S29 Wafer-scale characterization results for metalens imaging of wafer #T5 in reversed (back-to-front) configuration. (a) Layout with labelling convention of metalenses on wafer (front side image). (b,c) Broadband MWIR and LWIR images (no filter) using metalens and (d) images using hybrid metalens – BaF_2 configuration.

Figure S28 shows results for imaging of the tip of the soldering iron at 350ºC for the nine different metalenses on wafer #T5 with identical fabrication conditions to wafer #15 which was discussed in Section S4, with the individual lenses labelled according to Figure S28a. We measured the images of the metalens at MWIR and LWIR over the full spectral range, i.e. without any spectral filtering. Results are presented in Figure S28b and c. Additionally we measured the metalens in the hybrid configuration with the BaF_2 refractive lens as shown in Figure S28d.

Overall we see that all lenses are fully operational, with the highest transmitted intensity found for the central lens (0,0), consistent with the low alignment error in X and Y according

to Table S2. Importantly, the lenses further away from the center still show a good overall imaging quality with only an overall drop in absolute intensity observed. This result shows that the effect of front to back misalignment is moderate in the experimental metalenses even for large misalignments (the angular error of 0.09º for this wafer translates to a positional error >50 µm for the off-center lenses). Thus, the double-sided metalenses are fairly robust against misalignment in terms of functionality, but the best performance is expected for <10 µm alignment error. Using the same setup we also investigated the imaging capability of the metalenses when used in reversed configuration corresponding to its back-to-front orientation in the setup. Results are shown in Figure S29 and reveal a reasonably good imaging also for the reversed orientation.

S14. Removal of bad pixels and defects

In the main text, some minor defects in the images have been removed by replacing them with adjacent colors, as illustrated in Figure S30. No physical information is contained in these defects and all corrections made are cosmetic in nature. The cluster of around 10 dark pixels is an artefact from CO_2 laser damage on the camera produced by an overexposure incident during the focus measurements of Figure S17. Small arrows in the outdoor imaging test automatically generated in the imaging software were manually removed in the same way.

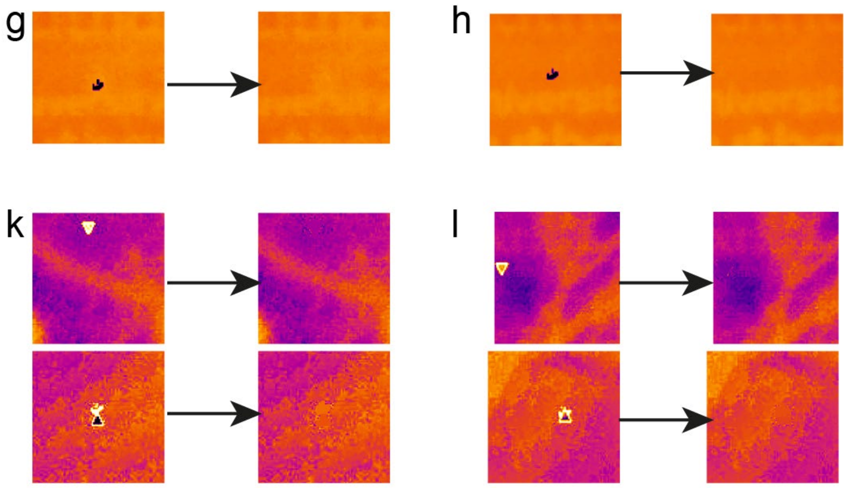

Figure S30 Details of images in main text Figure 4g, h, k, l before and after removal of bad pixels and markers.

References

(1) Zhao, F.; Zhao, C.; Zhang, Y.; Chen, J.; Li, S.; Zhou, W.; Ran, C.; Zeng, Y.; Chen, H.; He, X.; et al. Centimeter-size achromatic metalens in long-wave infrared. *Nanophotonics* **2025**, *14* (5), 589-599. DOI: doi:10.1515/nanoph-2024-0716.
(2) Dorrah, A. H.; Park, J.-S.; Palmieri, A.; Capasso, F. Free-standing bilayer metasurfaces in the visible. *Nature Communications* **2025**, *16* (1), 3126. DOI: 10.1038/s41467-025-58205-7.
(3) Vo, C.; Anderson, O.; Wirth-Singh, A.; Johnson, R.; Majumdar, A.; Coppens, Z. Broadband long-range thermal imaging via meta-correctors. *Appl. Opt.* **2025**, *64* (13), 3473-3479. DOI: 10.1364/AO.553334.
(4) Bosch, M.; Shcherbakov, M.; Won, K.; Lee, H.-S.; Kim, Y.; Shvets, G. Voltage-Tunable Multifunctional Zoom Imaging Metalenses. *ACS Photonics* **2025**, *12* (2), 728-736. DOI: 10.1021/acsphotonics.4c01530.
(5) Luo, Z.; Zhang, P.; Hou, H.; Li, Y.; Li, B.; Yi, Y.; Xu, L.; Meng, T.; Geng, Z.; Chen, M. K.; Zhao, Y. Colorimetric Thermography by a Long-Infrared Dual-Band Metalens. *Adv Sci (Weinh)* **2025**, *12* (2), e2408683. DOI: 10.1002/advs.202408683.
(6) Hou, M.; Chen, Y.; Li, J.; Yi, F. Single 5-centimeter-aperture metalens enabled intelligent lightweight mid-infrared thermographic camera. *Science Advances* **2024**, *10* (27), eado4847. DOI: doi:10.1126/sciadv.ado4847.
(7) Barulin, A.; Kim, Y.; Oh, D. K.; Jang, J.; Park, H.; Rho, J.; Kim, I. Dual-wavelength metalens enables Epi-fluorescence detection from single molecules. *Nat Commun* **2024**, *15* (1), 26. DOI: 10.1038/s41467-023-44407-4.
(8) Cao, G.; Wang, S.; Liu, W.; Zhang, H.; Han, J.; Xu, X.; Liu, J.; Zhao, W.; Li, H.; Lin, H.; et al. Two Hundred Nanometer Thin Multifocal Graphene Oxide Metalens for Varying Magnification Broadband Imaging. *ACS Nano* **2024**, *18* (52), 35550-35558. DOI: 10.1021/acsnano.4c13213.
(9) He, W.; Xin, L.; Yang, Z.; Li, W.; Wang, Z.; Liu, Z. Mid-infrared large-aperture metalens design verification and double-layer micro-optical system optimization. *Opt. Mater. Express* **2024**, *14* (5). DOI: 10.1364/ome.517520.
(10) Hu, T.; Wen, L.; Li, H.; Wang, S.; Xia, R.; Mei, Z.; Yang, Z.; Zhao, M. Aberration-corrected hybrid metalens for longwave infrared thermal imaging. *Nanophotonics* **2024**, *13* (17), 3059-3066. DOI: 10.1515/nanoph-2023-0918.
(11) Huang, L.; Han, Z.; Wirth-Singh, A.; Saragadam, V.; Mukherjee, S.; Froch, J. E.; Tanguy, Q. A. A.; Rollag, J.; Gibson, R.; Hendrickson, J. R.; et al. Broadband thermal imaging using meta-optics. *Nat Commun* **2024**, *15* (1), 1662. DOI: 10.1038/s41467-024-45904-w.
(12) Egede Johansen, V.; Gür, U. M.; Martínez-Llinás, J.; Fly Hansen, J.; Samadi, A.; Skak Vestergaard Larsen, M.; Nielsen, T.; Mattinson, F.; Schmidlin, M.; Mortensen, N. A.; Quaade, U. J. Nanoscale precision brings experimental metalens efficiencies on par with theoretical promises. *Communications Physics* **2024**, *7* (1). DOI: 10.1038/s42005-024-01598-6.
(13) Li, A.; Duan, H.; Jia, H.; Long, L.; Li, J.; Hu, Y. Large-aperture imaging system based on 100 mm all-Si metalens in long-wave infrared. *Journal of Optics* **2024**, *26* (6), 065005. DOI: 10.1088/2040-8986/ad3cf0.
(14) Lin, H.-I.; Geldmeier, J.; Baleine, E.; Yang, F.; An, S.; Pan, Y.; Rivero-Baleine, C.; Gu, T.; Hu, J. Wide-Field-of-View, Large-Area Long-Wave Infrared Silicon Metalenses. *ACS Photonics* **2024**, *11* (5), 1943-1949. DOI: 10.1021/acsphotonics.4c00013.
(15) Lin, R. J.; Su, V. C.; Wang, S.; Chen, M. K.; Chung, T. L.; Chen, Y. H.; Kuo, H. Y.; Chen, J. W.; Chen, J.; Huang, Y. T.; et al. Achromatic metalens array for full-colour light-field imaging. *Nat Nanotechnol* **2019**, *14* (3), 227-231. DOI: 10.1038/s41565-018-0347-0.
(16) Min, Q.; Trapp, J.; Fang, T.; Hu, R.; Wang, F.; Zhang, Z.; Liu, X.; Dai, A.; Yang, C.; Guo, J.; Situ, G. Varifocal Metalens for Compact and Accurate Quantitative Phase Imaging. *ACS Photonics* **2024**, *11* (7), 2797-2804. DOI: 10.1021/acsphotonics.4c00658.

(17) Moghaddasi, M.; Perez Coca, E. E.; Ye, D.; Flores, D. A.; Wu, X.; Jalal, A.; Ren, Z.; Abrinaei, F.; Hu, B. Wide FOV metalens for near-infrared capsule endoscopy: advancing compact medical imaging. *Nanophotonics* **2024**, *13* (24), 4417-4428. DOI: 10.1515/nanoph-2024-0393.
(18) Moon, S. W.; Kim, J.; Park, C.; Kim, W.; Yang, Y.; Kim, J.; Lee, S.; Choi, M.; Sung, H.; Park, J.; et al. Wafer-Scale Manufacturing of Near-Infrared Metalenses. *Laser & Photonics Reviews* **2024**, *18* (4). DOI: 10.1002/lpor.202300929.
(19) Park, J.-S.; Lim, S. W. D.; Amirzhan, A.; Kang, H.; Karrfalt, K.; Kim, D.; Leger, J.; Urbas, A.; Ossiander, M.; Li, Z.; Capasso, F. All-Glass 100 mm Diameter Visible Metalens for Imaging the Cosmos. *ACS Nano* **2024**, *18* (4), 3187-3198. DOI: 10.1021/acsnano.3c09462.
(20) Sharma, D. K.; Lai, K. H.; Baranikov, A. V.; Huang, A.; Lassalle, E.; Srivastava, S.; Loke, S.; Khaidarov, E.; Eschimese, D.; Fu, Y. H.; et al. Stereo Imaging with a Hemispherical Field-of-View Metalens Camera. *ACS Photonics* **2024**, *11* (5), 2016-2021. DOI: 10.1021/acsphotonics.4c00087.
(21) Sun, C.; Pi, H.; Kiang, K. S.; Yan, J.; Ou, J.-Y. Near-Infrared Metalens Empowered Dual-Mode High Resolution and Large FOV Microscope. *Advanced Optical Materials* **2024**, *n/a* (n/a), 2400512. DOI: 10.1002/adom.202400512.
(22) Xu, B.; Wei, W.; Tang, P.; Shao, J.; Zhao, X.; Chen, B.; Dong, S.; Wu, C. A Multi-foci Sparse-Aperture Metalens. *Adv Sci (Weinh)* **2024**, *11* (19), e2309648. DOI: 10.1002/advs.202309648.
(23) Zang, G.; Ren, J.; Shi, Y.; Peng, D.; Zheng, P.; Zheng, K.; Liu, Z.; Wang, Z.; Cheng, X.; Liu, A. Q.; Zhu, W. Inverse Design of Aberration-Corrected Hybrid Metalenses for Large Field of View Thermal Imaging Across the Entire Longwave Infrared Atmospheric Window. *ACS Nano* **2024**, *18* (49), 33653-33663. DOI: 10.1021/acsnano.4c12546.
(24) Zhang, J.; Sun, Q.; Wang, Z.; Zhang, G.; Liu, Y.; Liu, J.; Martins, E. R.; Krauss, T. F.; Liang, H.; Li, J.; Wang, X. H. A Fully Metaoptical Zoom Lens with a Wide Range. *Nano Lett* **2024**. DOI: 10.1021/acs.nanolett.4c00328.
(25) Badloe, T.; Kim, Y.; Kim, J.; Park, H.; Barulin, A.; Diep, Y. N.; Cho, H.; Kim, W. S.; Kim, Y. K.; Kim, I.; Rho, J. Bright-Field and Edge-Enhanced Imaging Using an Electrically Tunable Dual-Mode Metalens. *ACS Nano* **2023**, *17* (15), 14678-14685. DOI: 10.1021/acsnano.3c02471.
(26) Cheng, W.; Wang, Y.; Zhang, Y.; Chen, H.; Lu, Z.; Zhao, F.; Wang, Y.; Wu, J.; Yang, J. Broadband Achromatic Imaging of a Metalens with Optoelectronic Computing Fusion. *Nano Lett* **2024**, *24* (1), 254-260. DOI: 10.1021/acs.nanolett.3c03891.
(27) Go, G. H.; Park, C. H.; Woo, K. Y.; Choi, M.; Cho, Y. H. Scannable Dual-Focus Metalens with Hybrid Phase. *Nano Lett* **2023**, *23* (8), 3152-3158. DOI: 10.1021/acs.nanolett.2c04696.
(28) Kim, J.; Seong, J.; Kim, W.; Lee, G.-Y.; Kim, S.; Kim, H.; Moon, S.-W.; Oh, D. K.; Yang, Y.; Park, J.; et al. Scalable manufacturing of high-index atomic layer–polymer hybrid metasurfaces for metaphotonics in the visible. *Nature Materials* **2023**, *22* (4), 474-481. DOI: 10.1038/s41563-023-01485-5.
(29) McClung, A.; Torfeh, M.; Einck, V. J.; Watkins, J. J.; Arbabi, A. Visible Metalenses with High Focusing Efficiency Fabricated Using Nanoimprint Lithography. *Advanced Optical Materials* **2023**, *12* (9). DOI: 10.1002/adom.202301865.
(30) Vogliardi, A.; Ruffato, G.; Dal Zilio, S.; Bonaldo, D.; Romanato, F. Dual-functional metalenses for the polarization-controlled generation of focalized vector beams in the telecom infrared. *Sci Rep* **2023**, *13* (1), 10327. DOI: 10.1038/s41598-023-36865-z.
(31) Wang, Q.; Rogers, E. T. F.; Gholipour, B.; Wang, C.-M.; Yuan, G.; Teng, J.; Zheludev, N. I. Optically reconfigurable metasurfaces and photonic devices based on phase change materials. *Nature Photonics* **2016**, *10* (1), 60-65. DOI: 10.1038/nphoton.2015.247.
(32) Xu, P.; Li, X.; Yang, T.; Xiao, Y.; Cheng, X.; Lou, F.; Zhang, X.; Huang, H.; Zhang, X.; Wang, M.; et al. Long-infrared dual-wavelength linear-polarization-multiplexed confocal

metalens based on an all-silicon dielectric. *Opt Express* **2023**, *31* (16), 26685-26696. DOI: 10.1364/OE.494599.
(33) Zhang, L.; Chang, S.; Chen, X.; Ding, Y.; Rahman, M. T.; Duan, Y.; Stephen, M.; Ni, X. High-Efficiency, 80 mm Aperture Metalens Telescope. *Nano Letters* **2023**, *23* (1), 51-57. DOI: 10.1021/acs.nanolett.2c03561.
(34) Feng, W.; Zhang, J.; Wu, Q.; Martins, A.; Sun, Q.; Liu, Z.; Long, Y.; Martins, E. R.; Li, J.; Liang, H. RGB Achromatic Metalens Doublet for Digital Imaging. *Nano Lett* **2022**, *22* (10), 3969-3975. DOI: 10.1021/acs.nanolett.2c00486.
(35) Li, H.; Zhou, C.; Lee, W. B.; Choi, D. Y.; Lee, S. S. Flat telescope based on an all-dielectric metasurface doublet enabling polarization-controllable enhanced beam steering. *Nanophotonics* **2022**, *11* (2), 405-413. DOI: 10.1515/nanoph-2021-0609.
(36) Li, J.; Wang, Y.; Liu, S.; Xu, T.; Wei, K.; Zhang, Y.; Cui, H. Largest aperture metalens of high numerical aperture and polarization independence for long-wavelength infrared imaging. *Opt Express* **2022**, *30* (16), 28882-28891. DOI: 10.1364/OE.462251.
(37) Ryu, T.; Kim, M.; Hwang, Y.; Kim, M. K.; Yang, J. K. High-efficiency SOI-based metalenses at telecommunication wavelengths. *Nanophotonics* **2022**, *11* (21), 4697-4704. DOI: 10.1515/nanoph-2022-0480.
(38) Balli, F.; Sultan, M. A.; Ozdemir, A.; Hastings, J. T. An ultrabroadband 3D achromatic metalens. *Nanophotonics* **2021**, *10* (4), 1259-1264. DOI: 10.1515/nanoph-2020-0550.
(39) Huang, L.; Coppens, Z.; Hallman, K.; Han, Z.; Böhringer, K. F.; Akozbek, N.; Raman, A.; Majumdar, A. Long wavelength infrared imaging under ambient thermal radiation via an all-silicon metalens. *Opt. Mater. Express* **2021**, *11* (9), 2907-2914. DOI: 10.1364/OME.434362.
(40) Leitis, A.; Tseng, M. L.; John-Herpin, A.; Kivshar, Y. S.; Altug, H. Wafer-Scale Functional Metasurfaces for Mid-Infrared Photonics and Biosensing. *Adv Mater* **2021**, *33* (43), e2102232. DOI: 10.1002/adma.202102232.
(41) Lassalle, E.; Mass, T. W. W.; Eschimese, D.; Baranikov, A. V.; Khaidarov, E.; Li, S.; Paniagua-Dominguez, R.; Kuznetsov, A. I. Imaging Properties of Large Field-of-View Quadratic Metalenses and Their Applications to Fingerprint Detection. *ACS Photonics* **2021**, *8* (5), 1457-1468. DOI: 10.1021/acsphotonics.1c00237.
(42) Li, H.; Lee, W. B.; Zhou, C.; Choi, D. Y.; Lee, S. S. Flat Retroreflector Based on a Metasurface Doublet Enabling Reliable and Angle-Tolerant Free-Space Optical Link. *Advanced Optical Materials* **2021**, *9* (21). DOI: 10.1002/adom.202100796.
(43) McClung, A.; Samudrala, S.; Torfeh, M.; Mansouree, M.; Arbabi, A. Snapshot spectral imaging with parallel metasystems. *Science Advances* **2020**, *6* (38), eabc7646. DOI: doi:10.1126/sciadv.abc7646.
(44) Ndao, A.; Hsu, L.; Ha, J.; Park, J. H.; Chang-Hasnain, C.; Kante, B. Octave bandwidth photonic fishnet-achromatic-metalens. *Nat Commun* **2020**, *11* (1), 3205. DOI: 10.1038/s41467-020-17015-9.
